# Label-Free Proteomic Analysis of Protein Changes in the Striatum during Chronic Ethanol Use and Early Withdrawal

**DOI:** 10.3389/fnbeh.2016.00046

**Published:** 2016-03-11

**Authors:** Jennifer R. Ayers-Ringler, Alfredo Oliveros, Yanyan Qiu, Daniel M. Lindberg, David J. Hinton, Raymond M. Moore, Surendra Dasari, Doo-Sup Choi

**Affiliations:** ^1^Neurobiology of Disease PhD Program, Mayo Graduate School, Mayo ClinicRochester, MN, USA; ^2^Department of Molecular Pharmacology and Experimental Therapeutics, Mayo Graduate School, Mayo Clinic College of MedicineRochester, MN, USA; ^3^Department of Biochemistry and Molecular Biology, Center for Individualized Medicine, Mayo ClinicRochester, MN, USA; ^4^Department of Health Sciences Research, Mayo Clinic College of MedicineRochester, MN, USA; ^5^Department of Psychiatry and Psychology, Mayo Clinic College of MedicineRochester, MN, USA

**Keywords:** glutamate, addiction, alcohol, ethanol, withdrawal, chronic intermittent ethanol, proteomics, striatum

## Abstract

The molecular mechanisms underlying the neuronal signaling changes in alcohol addiction and withdrawal are complex and multifaceted. The cortico-striatal circuit is highly implicated in these processes, and the striatum plays a significant role not only in the early stages of addiction, but in the developed-addictive state as well, including withdrawal symptoms. Transcriptional analysis is a useful method for determining changes in gene expression, however, the results do not always accurately correlate with protein levels. In this study, we employ label-free proteomic analysis to determine changes in protein expression within the striatum during chronic ethanol use and early withdrawal. The striatum, composed primarily of medium spiny GABAergic neurons, glutamatergic and dopaminergic nerve terminals and astrocytes, is relatively homogeneous for proteomic analysis. We were able to analyze more than 5000 proteins from both the dorsal (caudate and putamen) and ventral (nucleus accumbens) striatum and identified significant changes following chronic intermittent ethanol exposure and acute (8 h) withdrawal compared to ethanol naïve and ethanol exposure groups respectively. Our results showed significant changes in proteins involved in glutamate and opioid peptide signaling, and also uncovered novel pathways including mitochondrial function and lipid/cholesterol metabolism, as revealed by changes in electron transport chain proteins and RXR activation pathways. These results will be useful in the development of novel treatments for alcohol withdrawal and thereby aid in recovery from alcohol use disorder.

## Introduction

The cortico-striatal circuit is highly implicated in both substance addiction and withdrawal. The different regions of this circuit are being intensely studied and extensive gene expression analyses have been performed (Bell et al., 2006; Melendez et al., 2012; Osterndorff-Kahanek et al., 2013, 2015; Most et al., 2015), effectively informing future research directions to aid in the development of potential therapeutics. However, gene expression does not always correlate with protein levels, especially during acute homeostatic changes, such as those occurring during early withdrawal. In fact, previous work has demonstrated that rapid modulation of synaptic protein expression may occur primarily via local translation of dendritic and axonal mRNA (Aguilar-Valles et al., 2015; Hussain and Bashir, 2015). Furthermore, ethanol may directly affect the activity or degradation of numerous proteins due to its lipophilic interaction with the plasma membrane and lipid rafts (Collin et al., 2014; Tang et al., 2014; Huang et al., 2016). The complexity of neuronal circuits involved in addiction and the multiphasic nature of addiction and withdrawal make it likely that changes in protein levels and/or function during addiction are both spatially and temporally dependent. Therefore, we performed a total protein analysis using label-free proteomics on the entire striatum of C57BL/6J mice undergoing chronic intermittent ethanol (CIE) exposure or acute withdrawal. This quantitative method of measuring protein levels provides a comprehensive and accurate depiction of real-time molecular events without relying on substrate labeling procedures. By comparing this systematic analysis of protein expression to previous transcriptional studies, the underlying mechanisms behind dysfunctional signaling in multiple regions of the striatum during various phases of addiction and withdrawal may be more accurately determined.

Research into the neuronal mechanisms of addiction has revealed changes primarily in the dopamine (DA), gamma-aminobutyric acid (GABA) and glutamate signaling pathways (Koob and Volkow, 2010; Koob, 2014; Most et al., 2014; Wang et al., 2014, 2015; Volkow et al., 2015; Didone et al., 2016). These changes are induced not only by the substance of abuse, but also by the context of conditioning (learning and memory), resulting in complex and variable neuroplastic changes occurring both dependently and independently of the substance of abuse (Berke and Hyman, 2000; Cunningham et al., 2006; Lobo and Nestler, 2011; Nam et al., 2013). One way of minimizing neuronal effects of the contextual and behavioral components of addiction in order to focus on the direct effects of alcohol is through passive ethanol exposure. Accordingly, the CIE procedure has been shown to reliably generate alcohol dependence in animals (Becker and Lopez, 2004; Lopez and Becker, 2005; DePoy et al., 2013; Meinhardt and Sommer, 2015) and produce severe withdrawal symptoms including ethanol withdrawal seizures, which peak 8 h after ethanol removal in mice as previously reported (Becker et al., 1997; Kim et al., 2011; Maldonado-Devincci et al., 2014; Meinhardt and Sommer, 2015), making it a powerful method for studying neuronal signaling during withdrawal.

It has been shown that alcohol dependence is caused by neuroadaptation to prolonged and excessive consumption of alcohol, a CNS depressant (Tsai et al., 1998). This dependence can lead to severe withdrawal symptoms upon cessation of alcohol consumption, termed alcohol withdrawal syndrome (AWS). AWS, which manifests with symptoms ranging from nausea and insomnia to hallucinations and seizures, may result in death due to severe symptoms associated with status epilepticus during the first 24 h of withdrawal in people (Bayard et al., 2004; Campos et al., 2011). These symptoms appear to result from increased neuronal excitability as a consequence of reduced GABA activity (Crews et al., 2013) and increased glutamatergic neurotransmission (Tsai et al., 1998). AWS is not only a medical emergency, but early symptoms can produce extreme discomfort increasing the drive to drink and impeding successful recovery from alcohol use disorder (AUD). Therefore, the development of therapeutics aimed at reducing or eliminating AWS is crucial for effective recovery from AUD.

The molecular mechanisms underlying the maladaptive neuronal signaling found in AUD and AWS are multifaceted, and remain undetermined. The dorsal (caudate and putamen, CPu) and ventral (nucleus accumbens, NAc) striatum play significant roles not only during early phases of addiction, but also in the post-addictive state (Chen et al., 2011), contributing to relapse and withdrawal (Cuzon Carlson et al., 2011). The CPu is a fairly homogenous region of the corticostriatal circuitry, composed primarily of medium spiny GABAergic neurons, glutamatergic nerve terminals, and astrocytes, making it a good candidate for such an expansive proteomic analysis technique. In contrast, the NAc is highly involved in motivational and contextual learning as well as the integration of environmental stimuli, and is more cellularly heterogeneous, yielding a less unified and perhaps more variable analysis (Koob and Volkow, 2010; Lobo and Nestler, 2011; Hinton et al., 2014). The results of proteomic changes in both regions are reported here for a comprehensive analysis of the effects of CIE exposure and acute withdrawal on the protein levels in the entire striatum.

## Materials and methods

### Animals

Male C57BL/6J mice (6 weeks old, Jackson Laboratories, Bar Harbor, ME) were grouped housed in standard Plexiglas cages under a 12 h light/dark cycle with lights on at 6:00 a.m. Food and water were provided *ad libitum*. Animal care and handling procedures were approved by the Mayo Clinic Institutional Animal Care and Use Committee (IACUC) in accordance with National Institutes of Health guidelines. At 8 weeks of age mice were randomly assigned to either ethanol or saline/air control groups. One cohort was used for proteomic analyses (*n* = 4–5 mice per group), while a separate cohort was used for behavioral testing (*n* = 9–10 mice per group).

### Chronic intermittent ethanol administration

**Figure 1:**
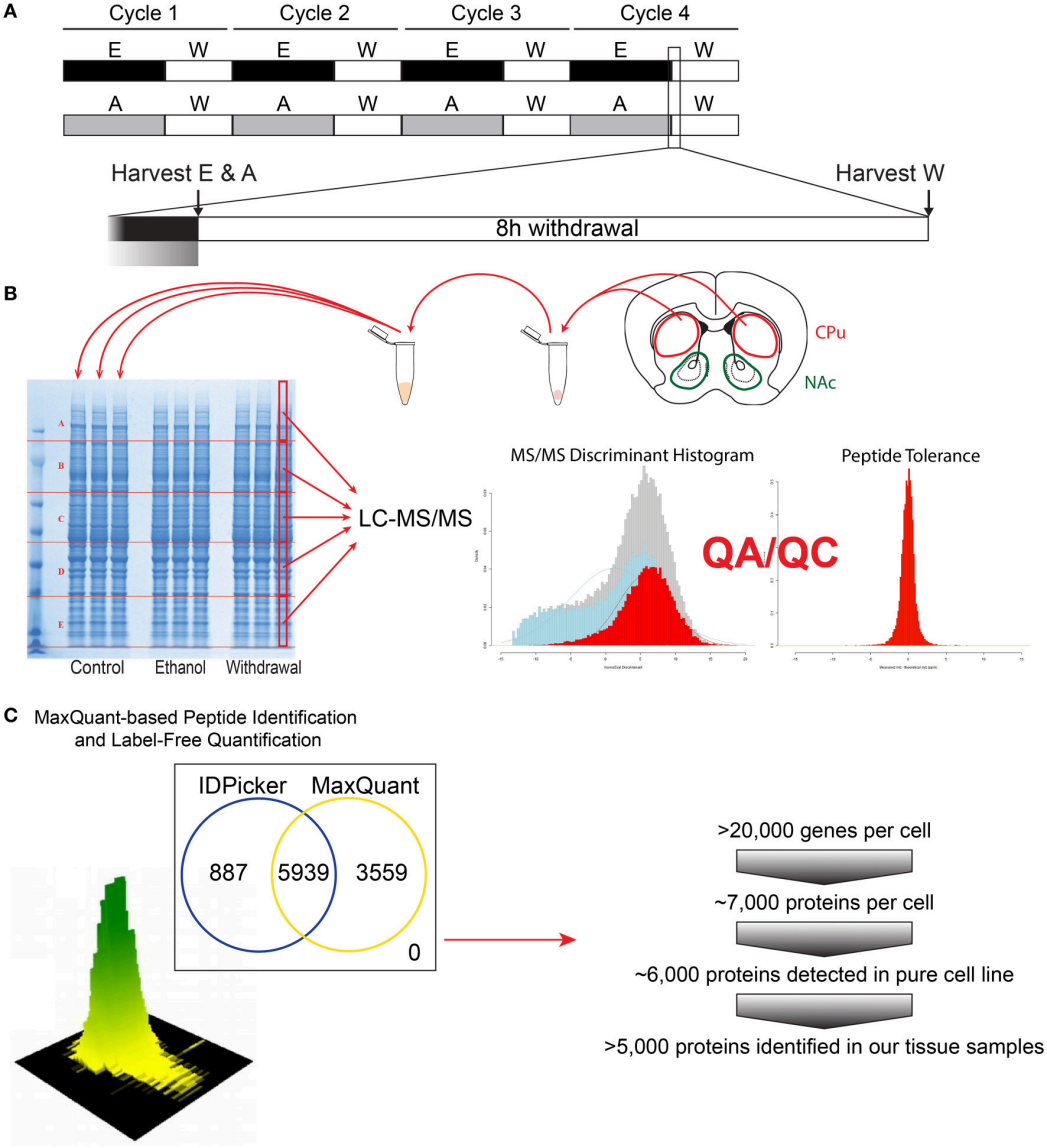
**Flow-chart of the experimental steps for proteomic analysis**. The CIE protocol and tissue harvest time points are depicted **(A)**. Tissue dissection (caudate and putamen shown for demonstration), protein extraction and subsequent proteomic analysis steps are shown **(B)**, followed by bioinformatics and protein identification steps **(C)**.

The CIE paradigm has been described in detail elsewhere (Becker and Lopez, 2004). Briefly, mice were exposed to ethanol vapor or room air using vapor administration chambers (La Jolla Alcohol Research, Inc., La Jolla, CA) for 16 h during the dark phase, followed by 8 h of room air in their home cages during the light phase. This process was repeated for 4 consecutive days, followed by 3 days in their home cages with room air (withdrawal period). This 7-day procedure entails one CIE cycle. We chose to use four CIE cycles (Figure 1A), as it has been shown to increase handling induced seizures and ethanol consumption in mice (Metten et al., 2010; DePoy et al., 2013). Ethanol was vaporized by pumping 95% ethanol into a heated flask to mix the vaporized ethanol with room air, continuously pumping the mixture into the chambers to maintain a concentration of 3.0–5.0 mg ethanol/liter of air. Air control chambers received identical airflow rates from room air. Prior to each vapor exposure, mice undergoing ethanol treatment were administered 1.5 g/kg ethanol (20% v/v in 0.9% saline) and 68.1 mg/kg pyrazole (SigmaAldrich, St. Louis, MO) in a single intraperitoneal (i.p.) injection to help initiate ethanol intoxication and maintain stable blood ethanol concentrations (BACs), respectively (Becker et al., 1997). Saline/air control mice received equal volume i.p. injections of 68.1 mg/kg pyrazole dissolved in 0.9% saline. After injections, mice were immediately placed inside the vapor chambers with food and water *ad libitum*. These procedures yielded consistent BACs of approximately 200 mg/dL for the duration of the CIE procedure.

### Blood alcohol concentration analysis

Mouse blood (~30 μL) was collected from tail veins with heparinized capillary tubes during the final hour of the fourth ethanol treatment each week for BAC analysis. Plasma BAC was determined using the Analox AM1 system (Analox Instruments USA, Lunenburg, MA). Statistical significance was determined by one-way ANOVA (for overall group BACs) or two-way ANOVA (for individual BACs) followed by Tukey *post-hoc* analysis for multiple comparisons.

### Ethanol drinking

Mice were tested for preference and consumption of 10% ethanol before and after CIE using the two-bottle continuous access, free-choice drinking test. Before CIE exposure, mice were given unlimited access to two bottles for 8 days. For the first 4 days, both bottles contained tap water to train the animals and to establish overall fluid consumptions. For the last 4 days, one bottle contained 10% ethanol and the other contained tap water. Initially and every 2 days thereafter at the same time of day, mice and bottles were weighed, and bottle positions were alternated to control for side preference.

After CIE, ethanol drinking was tested again for 14 days. Mice were individually housed for the duration of the drinking tests with *ad libitum* access to food. Data were analyzed by two-way repeated measures ANOVA with Bonferroni *post-hoc* tests.

### Loss of righting reflex

Fourteen days after the end of the final CIE cycle, mice were tested for loss of righting reflex (LORR). Mice were injected with a high dose of ethanol (i.p., 3.6 gm/kg; 20% (v/v) mixed in isotonic saline), and were intermittently placed on their backs in a v-shaped trough until they lost the ability to return to an upright position on all four paws. Time to recovery of LORR was measured and operationally defined as the length of time from initial LORR to when the mouse could right itself onto all four paws three times within a 30 s interval. Data were analyzed by unpaired two-tailed Student *t*-tests.

### Tissue collection and protein extraction

Mice receiving ethanol treatments were randomly divided into chronic ethanol (E) and early withdrawal (W) groups. E and saline/air (C) control mice were sacrificed at the end of the final 16 h of vapor exposure for tissue collection. W mice were removed from the vapor chambers at the same time point and blood was immediately collected for BAC analysis. W mice remained in their home cages in room air for 8 h before being sacrificed for tissue collection (Figure 1A).

Mice were anesthetized using carbon dioxide and the brains were immediately removed and washed in ice-cold PBS. Trunk blood was collected concurrently using heparinized capillary tubes for BAC analysis. The dorsal striatum was dissected from both hemispheres and immediately frozen on dry ice. To extract total protein, 0.5 mm zirconium oxide beads and 50 μL of ice-cold lysis buffer [CelLytic MT lysis reagent (Sigma-Aldrich), complete protease inhibitor cocktail (Roberts, Roche et al.) and phosphatase inhibitor cocktails type 2 and 3 (Sigma-Aldrich)] were added to the tissues. Tissues were homogenized in a Storm 24 Bullet Blender (Next Advance Inc, Averill Park NY, USA) for 4 min at 4°C and speed 4. Homogenates were centrifuged at 16,400 rpm at 4°C for 15 min. Supernatants were collected and analyzed for protein concentrations using Bradford Reagent (Bio-Rad). Equal amounts of protein from each animal for each group (*n* = 4) were pooled for subsequent denaturing electrophoresis (SDS-PAGE) (Figure 1B).

### Protein preparation for LC-MS/MS

Protein concentrations of the pooled protein samples were confirmed, and equal amounts of protein were denatured by boiling in NuPAGE®LDS Sample Buffer (Invitrogen, Carlsbad, CA) for 10 min at 70°C. Denatured lysates were loaded in triplicate (15 μg per lane) and resolved on a 4–12% Bis-Tris Gel (Figure 1B) in MOPS running buffer (Invitrogen, Carlsbad, CA). Gels were fixed with 50% methanol in 10% acetic acid, washed in ultra-pure water, then stained with Bio-Safe Coomassie Stain (Bio-Rad, Hercules, CA) per manufacturer's instructions. Gel lanes were then divided into 5 evenly spaced horizontal regions using protein bands that were common to all samples as guides. The middle half of each sample lane was cut length-wise. Gel sections were digested with trypsin following a previously described protocol (Hogan et al., 2015). In brief, each gel section was de-stained, reduced with dithiothreitol and alkylated with iodoacetamide. Proteins were digested overnight at 37°C with 140 ng of trypsin dissolved in 25 mM Tris (pH 8.2). Peptides were extracted from the gel piece with 50% acetonitrile (ACN) in 4% trifluoroacetic acid (TFA), followed by two additional extractions with ACN. The combined extracts were evaporated to dryness on a vacuum concentrator and stored at −80°C until further analysis.

### Liquid chromatography-tandem mass spectrometry (LC-MS/MS) analysis

Peptide extract from each gel section (see Figure 1B) was reconstituted in 40 μL HPLC-grade water containing 0.2% formic acid (FA), 0.1% TFA, and 0.002% Zwittergent 3–16. 10 μL of the peptide extract (15 μL for the two higher molecular weight gel sections) were loaded onto a 0.25 μL bed OptiPak trap (Optimize Technologies, Oregon City, Oregon) custom-packed with 5 μm, 200Å Magic C8 (Bruker-Michrom, Auburn, CA) stationary phase. Loaded trap was washed for 4 min with an aqueous loading buffer of 0.2% FA and 0.05% TFA at 10 μL /min. Following the wash, peptides were transferred via 10-port valve onto a 35 cm × 100 μm PicoFrit column 9 (NewObjective, Woburn, MA), self-packed with Agilent Poroshell 120S 2.7 μm EC-C18 stationary phase, using a Dionex UltiMate® 3000 RSLC liquid chromatography (Ferrer-Alcón et al., 2003) system (Thermo-Fisher Scientific, Waltham, MA). Peptides were separated using a 400 nL/min LC gradient comprised of 2–30%B in 0–70 min, 30–50%B from 70 to 100 min, 50–95%B from 100 to 104 min, held at 95%B for 8 min and re-equilibrated to 2%B. Mobile phase A was 2% ACN in water with 0.2% FA and mobile phase B was ACN/isopropanol/water (80/10/10 by volume) with 0.2% FA. Eluting peptides were analyzed using a QExactive mass spectrometer (Thermo-Fisher Scientific, Waltham, MA). The instrument was operated in data-dependent mode by collecting MS1 data at 70,000 resolving power (measured at m/z 200) with an AGC value of 3E6 over an m/z range of 350–2000, using lock masses from background polysiloxanes at m/z 371.10123 and 445.12002. Precursors were fragmented with normalized collision energy of 27, fragments measured at 17,500 resolving power and a fixed first mass of 140. Tandem mass spectra (MS/MS) were collected on the top 15 precursor masses present in each MS1 using an AGC value of 1E5, max ion fill time of 100 ms, an isolation window of 3.0 Da, isolation offset of 0.5 Da, and a dynamic exclusion time of 60s.

### Bioinformatic analysis of LC-MS/MS data

We utilized a label-free peptide MS1 intensity-based method for finding differentially expressed proteins between experimental groups (Figure 1C). The quality of the raw data was assessed using the quality control metrics in the Swift proteomic data processing pipeline (Zenka et al., 2011). MaxQuant (version 1.5.1) software processed the raw data files to produce a list of protein groups and their corresponding intensities in each sample (Cox et al., 2014). To accomplish this, MaxQuant was configured to use a composite mouse protein sequence database containing UniProt mouse reference proteome (downloaded on 12 February 2015) and sequences of common contaminants (trypsin, keratin, cotton, wool, etc.). Reversed protein sequences were appended to the database for estimating protein identification false discovery rates (FDRs). The software was configured to use 20 ppm m/z tolerance for precursors and fragments while performing peptide-spectrum matching. The software derived semitryptic peptides from the sequence database while looking for the following variable modifications: carbamidomethylation of cysteine (+57.023 Da.), oxidation of methionine (+15.994 Da.), formation of n-terminal pyroglutamic acid (−17.023 Da.) and protein n-terminal acetylation (+42.01 Da.). MaxQuant was instructed to align the runs and match features between multiple sample runs of the same gel region. The software filtered peptide and protein identifications at 2% FDR, grouped protein identifications into groups and reported protein group intensities.

An in-house script written in R programming language performed differential expression analysis using protein group intensities. First, protein group intensities of each sample were log2 transformed and normalized using Quantile method. For each protein group, the normalized intensities observed in two groups of samples were modeled using a Gaussian-linked generalized linear model. An ANOVA test was used to detect the differentially expressed protein groups between pairs of experimental groups. Differential expression *p*-values were FDR corrected using Benjamini-Hochberg-Yekutieli procedure. Protein groups with an FDR < 0.05 and an absolute log2 fold change of at least 0.5 were considered as significantly differentially expressed and saved for pathway analysis.

### Proteomic data analysis using ingenuity pathway analysis software

QIAGEN's Ingenuity® Pathway Analysis (IPA®, QIAGEN Redwood City, CA, USA; www.qiagen.com/ingenuity) was used to analyze the proteins identified as significantly different by the bioinformatics described above. Proteins were further restricted to include only those with intensity value readings from all three technical replicates. The normalized ratios, *p*-values and FDRs from these resulting proteins were uploaded to IPA with their corresponding SwissProt/UniProt identifiers. IPA was then instructed to only analyze proteins with equal to or greater than a log2 fold change of 1.25, corrected *p*-value of less than 0.05, and a FDR (*q*-value) of 0.001. IPA Knowledge Base was restricted to select tissues and cells primarily from the nervous and immune systems (see Supplemental Data sheet 1 for CPu, and Data sheet 8 for NAc for IPA settings). IPA used the right tailed Fisher's exact test to determine statistical significance. Results are given with significance (*p*-value), ratio ([number of proteins from data set]/[total known proteins in pathway]), and/or z-score (number of standard deviations above or below the mean) when applicable.

## Results

### BAC and ethanol consumption

**Figure 2:**
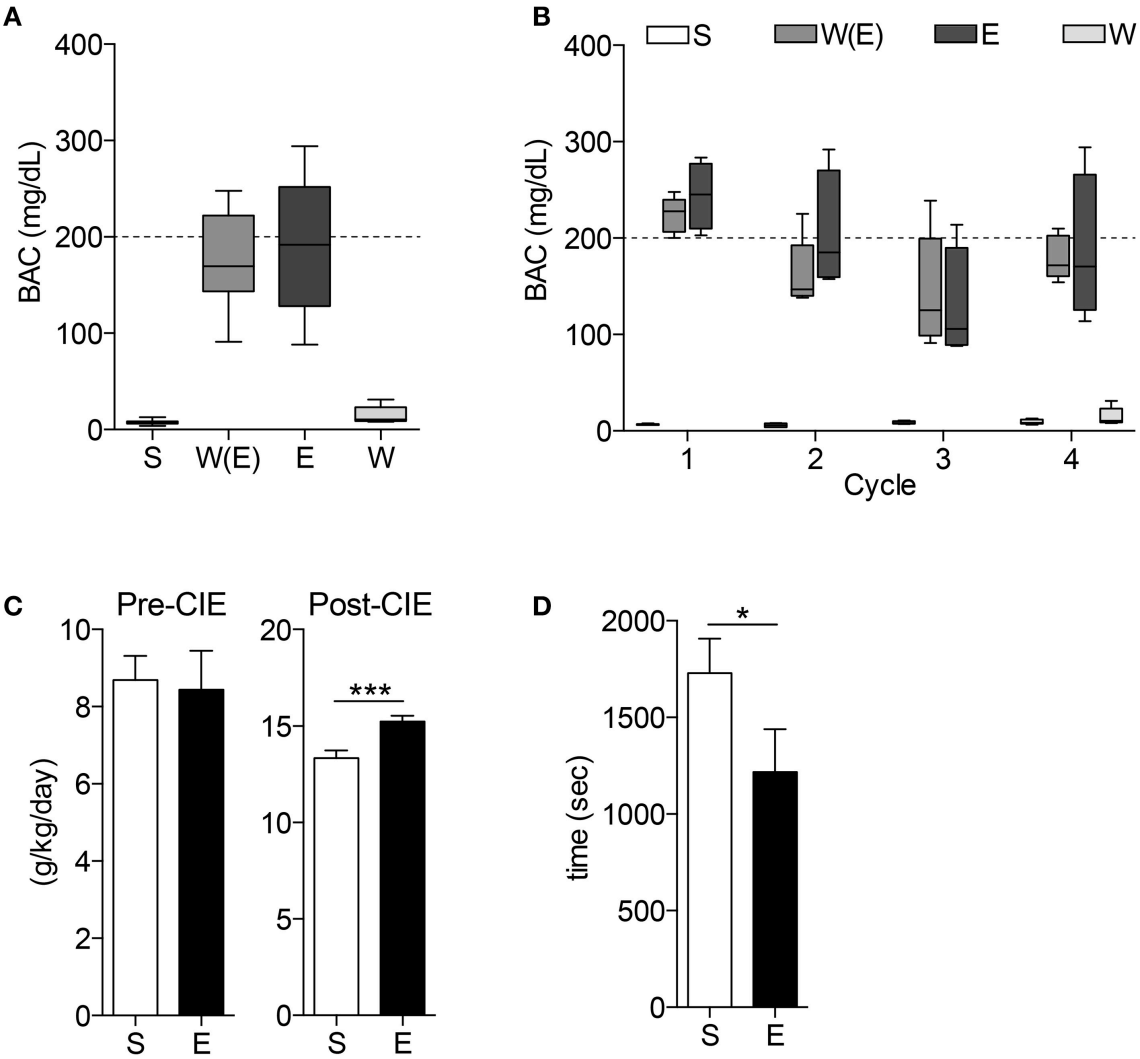
**Blood alcohol concentrations and behavior tests demonstrate animals display alcohol-dependent phenotype**. The blood alcohol concentrations (BAC) of the mice are within the optimal range for developing alcohol dependence throughout the experiment **(A)** and during each cycle **(B)**. 10% ethanol consumption **(C)** is significantly higher in E mice after CIE treatments (^***^p < *0.001*). CIE mice also demonstrate shorter time to recovery during loss of righting reflex (LORR) test (**D**, ^*^p < *0.05*).

All mice receiving ethanol treatments had the same BAC of approximately 200 mg/dL for the duration of the CIE procedure, and at the time of tissue harvest W mice BACs were no different than C mice (Figures 2A,B. *n* = 4–5 per group). After the CIE procedure, mice exposed to ethanol consumed significantly more 10% ethanol during two-bottle choice than mice exposed to room air (Figure 2C. *n* = 9–10 per group, ^***^p < *0.001*). These mice also had a faster time to recovery from loss of righting reflex than the air control group (Figure 2D. *n* = 9–10 per group, ^*^p < *0.05*).

### Label-free proteomic analysis

LC-MS/MS analysis (Figures 1B,C) detected between 5085 and 5944 unique proteins from each comparison between the three treatment groups (CPu: E vs. C = 5085; W vs. E = 5086; W vs. C = 5086; NAc: E vs. C = 5940; W vs. E = 5944; W vs. C = 5942). After exclusion of non-triplicate values, up to 5672 proteins from each comparison were uploaded to IPA for differential expression analysis (CPu: E vs. C = 4896; W vs. E = 4895; W vs. C = 4893; NAc: E vs. C = 5648; W vs. E = 5672; W vs. C = 5651). IPA determined that up to 284 proteins were significantly altered in each of the three comparisons (CPu: E vs. C = 168; W vs. E = 157; W vs. C = 284; NAc: E vs. C = 148; W vs. E = 231; W vs. C = 118) under the more restrictive exclusion criteria. (Complete data sets can be found in Supplemental Materials. CPu data for E vs. C in Data sheet 2, W vs. E in Data sheet 3, and W vs. C in Data sheet 4. NAc data for E vs. C in Data sheet 5, W vs. E in Data sheet 6, and W vs. C in Data sheet 7).

From this point forward, this manuscript will focus on the E vs. C (“E” mice) and W vs. E (“W” mice) groups in an effort to remain focused on pathways of pre-clinical importance.

### Top affected canonical pathways

**Figure 3:**
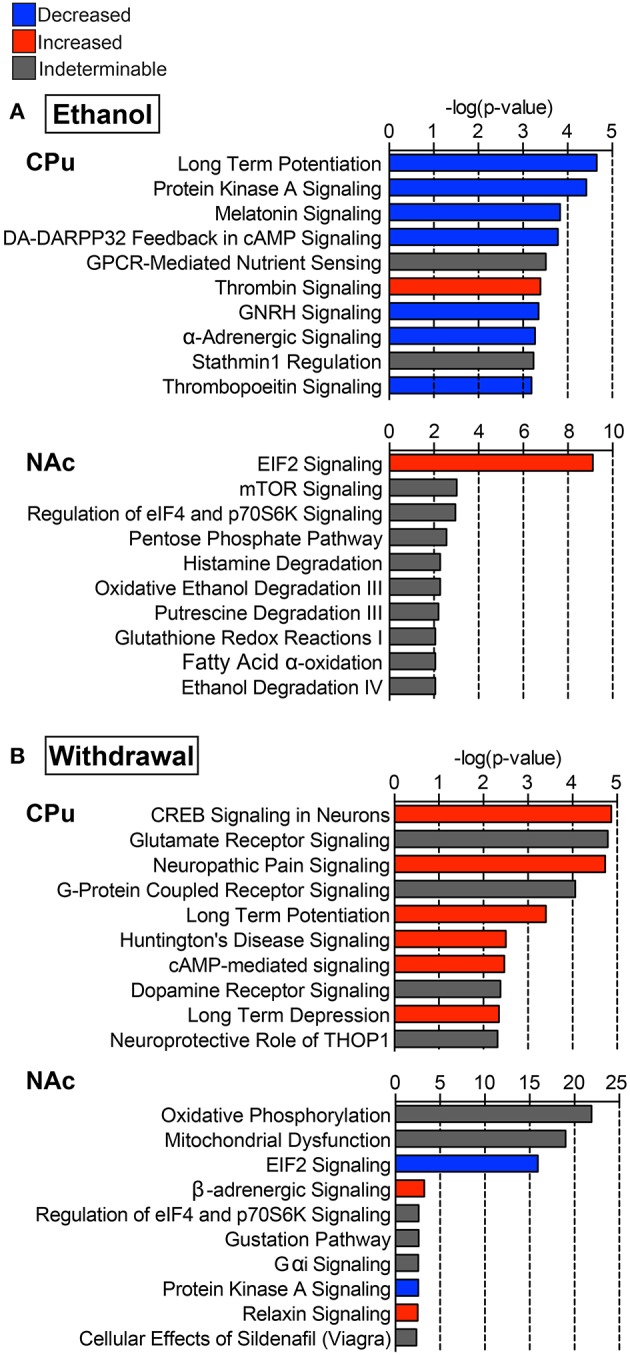
**Select top significant canonical pathways**. Ten of the top canonical pathways as determined by *p*-value of overlap between pathway molecules and our data sets are shown for both **(A)** ethanol (E vs. C group) and **(B)** withdrawal (W vs. E group) in the caudate-putamen (“CPu”) and nucleus accumbens (“NAc”). Blue indicates negative, and red indicates positive overall z-scores; gray indicates that a pattern could not be determined. Complete data can be found in Supplemental Materials.

IPA identified hundreds of canonical pathways as significantly affected in each experimental comparison. The top 10 canonical pathways, as ranked by *p*-value, are shown in Figure 3. When considering z-scores, an overall trend of decreased activity in the E mice, and increased activity in W mice is evident for the CPu data. Following this trend, many pathways were differentially activated in both striatal regions between the two treatment groups. In the CPu, for example synaptic plasticity was highly significantly and differentially altered. In E mice, long-term potentiation (LTP) was the most significantly affected canonical pathway, with a relatively large decrease (Figure 3A, z-score = −1.633) in overall activity, while in W mice activity was slightly increased (Figure 3B, z-score = 0.82). Long-term depression (LTD), on the other hand, was significantly increased in W mice (Figure 3B, z-score = 1.34), but activity change was far less significant in E mice (*p* = 0.07) and direction of change could not be determined. In contrast, eIF2 signaling, which is related to mRNA translation, was differentially activated in the opposite direction in the NAc, being increased in E mice (Figure 3A, z-score = 2.83) but decreased in W mice (Figure 3B, z-score = −4), and with far greater significance than any of the pathways in the CPu (E *p*-value = 7.76E-10; W *p*-value = 1.26E-16).

In the CPu, cAMP response element binding protein (CREB)-related and PKA signaling pathways were among the top affected canonical pathways. In E mice, activity in these pathways was decreased (Figure 3A, z-scores for PKA = −1, dopamine-DARPP32 feedback in cAMP = −2.646, CREB signaling in neurons = −1.633), while W mice showed increased activity in dopamine-DARPP32 feedback in cAMP (z-score = 1.00) and CREB signaling in neurons (Figure 3B, z-score = 0.71), being the most significantly affected canonical pathway in W mice (*p* = 1.35E-05). CREB signaling in neurons was also increased in the NAc during withdrawal (z-score = 1), but was not significantly altered in the NAc in E mice.

Glutamate receptor signaling was the second most significantly affected canonical pathway in the CPu of W mice (Figure 3B, *p* = 1.62E-05), sharing many of the same altered proteins as the top 7 affected pathways. In the NAc oxidative phosphorylation (Figure 3B, *p* = 1.26E-22) was the most significantly affected canonical pathway during withdrawal. Additionally, mitochondrial dysfunction was one of the top 20 significantly affected canonical pathways in W mice (*p*-values CPu = 1.02E-02, NAc = 1.00E-19; Figure 3B). Other significantly activated pathways in the CPu of W mice include FXR/RXR activation (*p* = 8.78E-03) and positive acute phase response proteins (*p* =1.39E-02; see Supplemental Data sheet 3), reflecting alterations in lipid metabolism and inflammatory response.

### Top altered proteins and upstream regulators

Based on our neuroproteomic examination and subsequent bioinformatic analysis, we were able to identify several key focus proteins that inherently had the greatest fold change expression in each comparison group. The top differentially expressed proteins found in the CPu and NAc are listed in Tables 1–4, respectively. Though many of the altered proteins in E mice were related to small molecule biochemistry and extracellular signaling, the more familiar addiction-associated proteins were seen in the withdrawal groups (Tables 2, 4), such as prodynorphin (PDYN), cannabinoid receptor 1 (CNR1) and calcium/calmodulin-dependent protein kinase IIα (CAMK2A).

**Table 1:** **Select top significantly altered analysis-ready molecules in the caudate-putamen of mice receiving chronic ethanol treatment (relative to control mice)**.

**Direction of change**	**Protein ID**	**Protein name**	**Accession number**	**Log2 Fold change**
UP	TRIM32	Tripartite motif containing 32; E3 ubiquitin ligase	Q8CH72	4.34E+10
	TLE4	Transducin-like enhancer of split 4	Q62441	1.03E+10
	LIAS	Lipoic acid synthetase	Q99M04	6.968
	SERPINC1	Serpin peptidase inhibitor, clade C (antithrombin), member 1	P32261	5.965
	GPX3	Glutathione peroxidase 3	P46412	4.745
	TTR	Transthyretin	P07309	4.582
	MICALL1	MICAL-like 1	Q8BGT6	4.460
	CX3CL1	Chemokine (C-X3-C motif) ligand 1	O35188	3.020
	TP53I11	Tumor protein p53 inducible protein 11	Q4QQM4	2.898
	SERPINF2	Serpin peptidase inhibitor, clade F (alpha−2 antiplasmin, pigment epithelium derived factor), member 2	Q61247	2.839
DOWN	BID	BH3 interacting domain death agonist	P70444	−7.634
	HAP1	Huntingtin-associated protein 1	O35668−2	−5.385
	HP	Haptoglobin	Q61646	−3.997
	PTPDC1	Protein tyrosine phosphatase domain containing 1	Q6NZK8−2	−3.799
	PLXNC1	Plexin C1	Q9QZC2	−3.310
	MYO1B	Myosin IB	P46735−2	−3.130
	AKAP2	A kinase (PRKA) anchor protein 2	O54931-5	−2.939
	RGS14	Regulator of G-protein signaling 14	P97492	−2.647
	WFS1	Wolfram syndrome 1 (wolframin)	P56695	−2.482
	DOCK10	Dedicator of cytokinesis 10	Q8BZN6	−2.353

**Table 2:** **Select top significantly altered analysis-ready molecules in the caudate-putamen of mice during acute withdrawal from chronic ethanol (relative to mice receiving chronic ethanol)**.

**Direction of change**	**Protein ID**	**Protein name**	**Accession number**	**Log2 Fold change**
UP	PDYN	Prodynorphin	O35417	8.682
	BID	BH3 interacting domain death agonist	P70444	8.642
	TMSB10	Thymosin beta 10	P62329	4.609
	CNR1	Cannabinoid receptor 1 (brain)	P47746	2.783
	COQ7	Coenzyme Q7 homolog, ubiquinone	P97478	2.745
	MYO1B	Myosin IB	P46735-2	2.635
	SLC6A3	Solute carrier family 6 (neurotransmitter transporter), member 3; NA^+^-dependent dopamine transporter	Q61327	2.497
	SLC1A3	Solute carrier family 1 (glial high affinity glutamate transporter), member 3; EAAT1, GLAST	P56564	2.370
	STK26	Serine/threonine protein kinase 26	Q99JT2	2.325
	WFS1	Wolfram syndrome 1 (wolframin)	P56695	2.252
DOWN	LIAS	Lipoic acid synthetase	Q99M04	−7.721
	RPL28	Ribosomal protein L28	P41105	−6.233
	CHERP	Calcium homeostasis endoplasmic reticulum protein	Q8CGZ0	−6.140
	SERPINC1	Serpin peptidase inhibitor, clade C (antithrombin), member 1	P32261	−6.102
	SERPINF2	Serpin peptidase inhibitor, clade F (alpha-2 antiplasmin, pigment epithelium derived factor), member 2	Q61247	−4.441
	KIAA1217	Sickle tail protein homolog	A2AQ25−4	−4.407
	ILF3	Interleukin enhancer binding factor 3, 90kDa	Q9Z1X4	−4.204
	TTR	Transthyretin	P07309	−4.120
	GPX3	Glutathione peroxidase 3	P46412	−3.330
	SERPINA3	Serpin peptidase inhibitor, clade A (alpha-1 antiproteinase, antitrypsin), member 3	P07759	−3.188

**Table 3:** **Select top significantly altered analysis-ready molecules in the nucleus accumbens of mice receiving chronic ethanol treatment (relative to control mice)**.

**Direction of change**	**Protein ID**	**Protein name**	**Accession number**	**Log2 Fold change**
UP	CENPE	Centromere protein E, 312kDa	E9QKK1	3.07E+10
	ITGA3	Integrin, alpha 3 (antigen CD49C, alpha 3 subunit of VLA-3 receptor)	Q62470-3	5.67E+09
	PDLIM4	PDZ and LIM domain 4	P70271	4.041
	CPLX3	Complexin 3	Q8R1B5	2.899
	HSPG2	Heparan sulfate proteoglycan 2	B1B0C7	2.867
	H3F3A/H3F3B	H3 histone, family 3A	E0CZ27	2.737
	DNMT1	DNA (cytosine-5-)-methyltransferase 1	J3QNW0	2.664
	CBLN1	Cerebellin 1 precursor	Q9R171	2.623
	ITIH2	Inter-alpha-trypsin inhibitor heavy chain 2	Q61703	2.595
DOWN	ADD1	Adducin 1 (alpha)	E9Q1K3	−5.359
	KCNQ3	Potassium channel, voltage gated KQT-like subfamily Q, member 3	Q8K3F6	−4.013
	GDPD5	Glycerophosphodiester phosphodiesterase domain containing 5	Q640M6	−3.503
	ELFN1	Extracellular leucine-rich repeat and fibronectin type III domain containing 1	Q8C8T7	−3.385
	LAMP5	Lysosomal-associated membrane protein family, member 5	Q9D387	−3.080
	MCTP1	Multiple C2 domains, transmembrane 1	E9PW38	−2.854
	SMAD2	SMAD family member 2	Q62432−2	−2.801
	KIRREL3	Kin of IRRE like 3 (Drosophila)	G5E8B6	−2.746
	APOB	Apolipoprotein B	E9Q1Y3	−2.568
	OTOF	Otoferlin	D3YXV0	−2.531

**Table 4:** **Select top significantly altered analysis-ready molecules in the nucleus accumbens of mice during acute withdrawal from chronic ethanol (relative to mice receiving chronic ethanol)**.

**Direction of change**	**Protein ID**	**Protein name**	**Accession number**	**Log2 Fold change**
UP	SLC35D3	Solute carrier family 35, member D3	Q8BGF8	7.436
	ZDHHC17	zinc finger, DHHC-type containing 17	Q80TN5	6.049
	WWP2	WW domain containing E3 ubiquitin protein ligase 2	Q9DBH0	5.844
	ADD1	Adducin 1 (alpha)	E9Q1K3	5.557
	ELFN1	Extracellular leucine-rich repeat and fibronectin type III domain containing 1	Q8C8T7	5.47
	FGFR3	Fibroblast growth factor receptor 3	Q61563	5.341
	ABCB11	ATP-binding cassette, sub-family B (MDR/TAP), member 11	J3QNY6	4.457
	GDPD5	Glycerophosphodiester phosphodiesterase domain containing 5	Q640M6	3.613
	LAMP5	Lysosomal-associated membrane protein family, member 5	Q9D387	3.531
	KCNQ3	Potassium channel, voltage gated KQT-like subfamily Q, member 3	Q8K3F6	3.201
DOWN	CAMK2A	Calcium/calmodulin-dependent protein kinase II alpha	F8WIS9	−7.31E+10
	PPM1L	Protein phosphatase, Mg2+/Mn2+ dependent, 1L	Q8BHN0	−3.48E+10
	AVP	Arginine vasopressin	P35455	−5.26E+08
	MOBP	Myelin-associated oligodendrocyte basic protein	Q9D2P8	−14.701
	H3F3A/H3F3B	H3 histone, family 3A	E0CZ27	−11.249
	CLDN11	Claudin 11	Q60771	−10.707
	CPLX3	Complexin 3	Q8R1B5	−7.681
	HIST1H1D	Histone cluster 1, H1d	P43277	−6.015
	H2AFV	H2A histone family, member V	Q3THW5	−5.9
	STX3	Syntaxin 3	Q64704-2	−4.468

**Figure 4:**
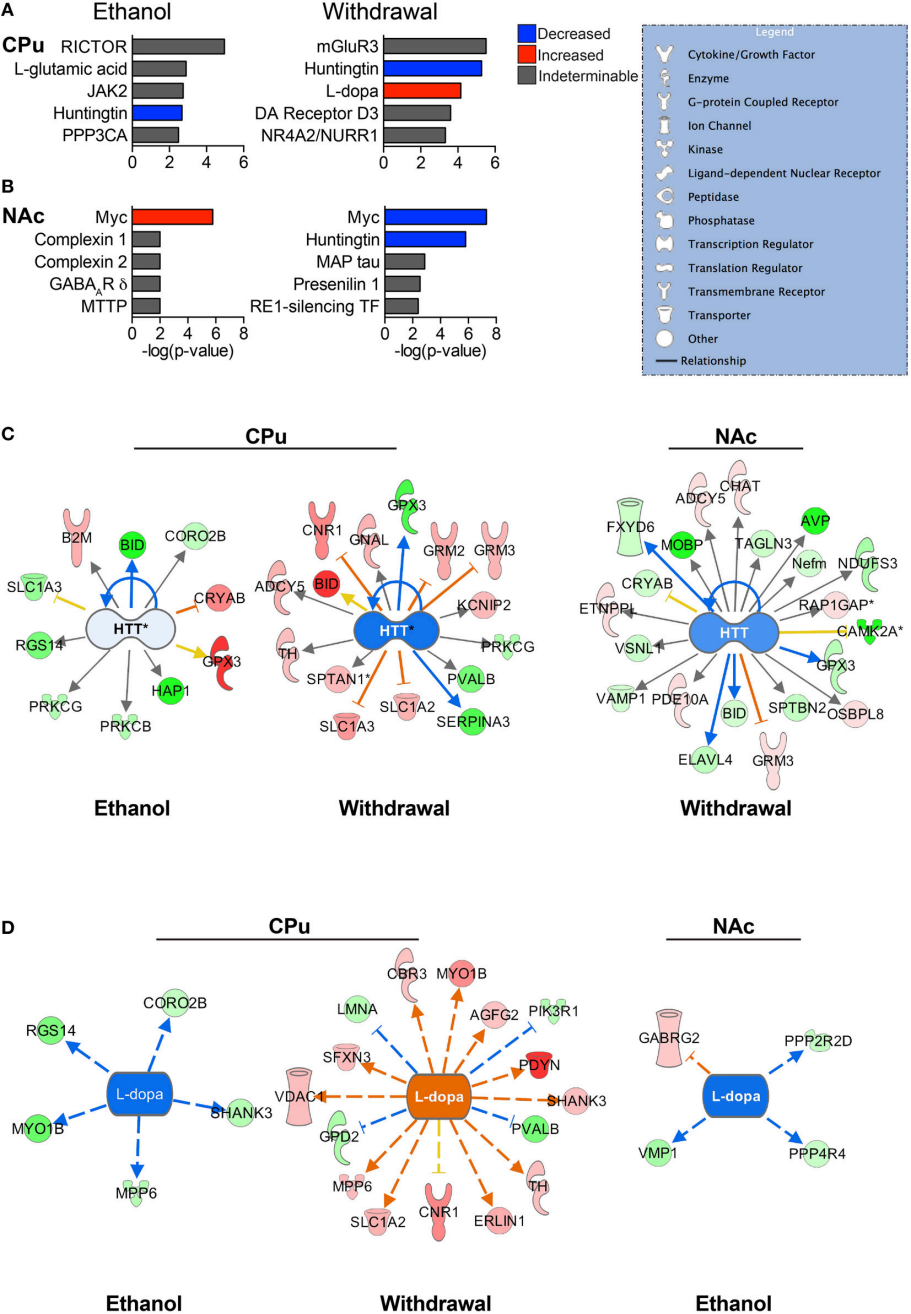
**Predicted upstream regulators**. The most significant predicted upstream regulators in the CPu **(A)** and NAc **(B)** are shown. Select pathways are represented to illustrate the differential protein expressions that led to the status of upstream regulation prediction. Huntingtin (HTT) **(C)** is one of the most significantly affected upstream regulators for both the CPu and the NAc, playing a major role in normal cellular maintenance. L-dopa **(D)** has a predicted decrease in activity during CIE, and increase during withdrawal. (Color key: blue/green = decreased/down-regulated; orange/red = increased/up-regulated; “DA” = dopamine). Complete data can be found in Supplemental Materials.

The top significant upstream regulators predicted by IPA are shown for the CPu (Figure 4A) and NAc (Figure 4B), with blue indicating negative z-scores and red indicating positive z-scores. Huntingtin (HTT) is a top predicted upstream regulator for both groups (Figure 4C) in all but the NAc of E mice. HTT appears to have reduced activity in all conditions, with a greater predicted reduced activity in W mice (z-score = −1.842 for CPu, −1.069 for NAc) than E mice (z-score = −0.106 for CPu). The transcriptional repressor, Myc (Figures 4A,B), however, shows far greater significance in the NAc than in the CPu, predicted to be activated in E mice (z-score = 1.897) and inhibited during withdrawal (z-score = −2.673). In contrast, Myc is predicted to be inhibited in the CPu of E mice (z-score = −0.447). Interestingly, L-dopa (Figure 4D) is predicted to have increased activity in the CPu of W mice (z-score = 3.664), and decreased activity in E mice (z-scores CPu = −2.236, NAc = −2.00), while dopamine is a predicted upstream regulator in the CPu of W mice, (*p* = 2.48E-02) though in an undeterminable direction. Also, based on changes in B2M, BCL2L1, and SLC1A3 protein levels in the CPu of E mice, L-glutamate (Figure 4A) is a predicted upstream regulator. Similarly, mGluR3 is predicted to be an important upstream regulator in the CPu during withdrawal based on changes in the levels of GRM2, SLC1A2, and SLC1A3 (see Supplemental Data sheet 3).

### Protein networks are differentially affected by CIE and withdrawal

**Figure 5:**
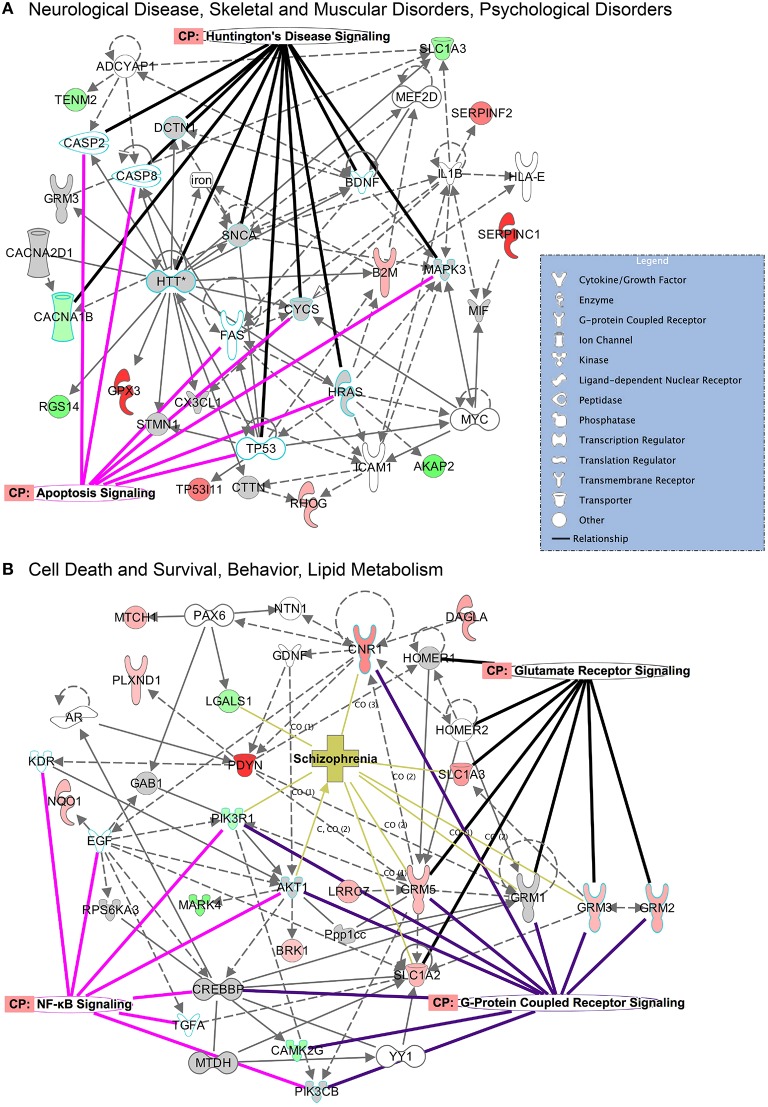
**Top networks activated in the CPu during CIE and acute withdrawal**. Illustrated here are select top network pathways for each condition in the CPu. **(A)** The network encompassing neurological disease, skeletal and muscular disorders, and psychological disorders is shown for the CIE mice, and **(B)** the network encompassing cell death and survival, behavior and lipid metabolism is shown for the withdrawal mice. Networks are overlaid with significantly affected canonical pathways and diseases. (Color key: green = down-regulated, red = up-regulated, gray = molecule present in dataset but not significantly changed, white = molecule important in pathway but not found in dataset; overlay colors are unique to each canonical pathway or disease). Complete data can be found in Supplemental Materials.

The top protein networks associated with our differentially expressed protein data set in E mice included cell morphology, cellular development, cellular growth and proliferation (CPu) and tissue development, cell death and survival, cardiovascular system development and function (Table S1). The top networks associated with the W mice data set included behavior, cell-to-cell signaling and interaction, drug metabolism and biliary hyperplasia, hepatic system development and function, liver cholestasis (Table S2). Both groups showed high associations with “cell-to-cell signaling and interaction” and “hereditary disorder” (Huntington's Disease), likely reflective of the tissue and cell types analyzed. Illustrated in Figure 5 are two of the top networks activated in the CPu for each condition. These networks are overlaid with top canonical pathways based on overlap *p*-values. Many of the top networks in the E mice involved molecular and cellular functions, highlighted by the apoptosis signaling and Huntington's disease signaling (encompassing many molecules necessary for normal neuronal function) overlays in Figure 5A. The network illustrated in Figure 5B reveals the interactions between behavioral signaling and lipid metabolism with cell death and survival pathways, with overlays highlighting proteins that are also involved in glutamate receptor signaling, GPCR signaling, NFκB signaling and schizophrenia.

**Figure 6:**
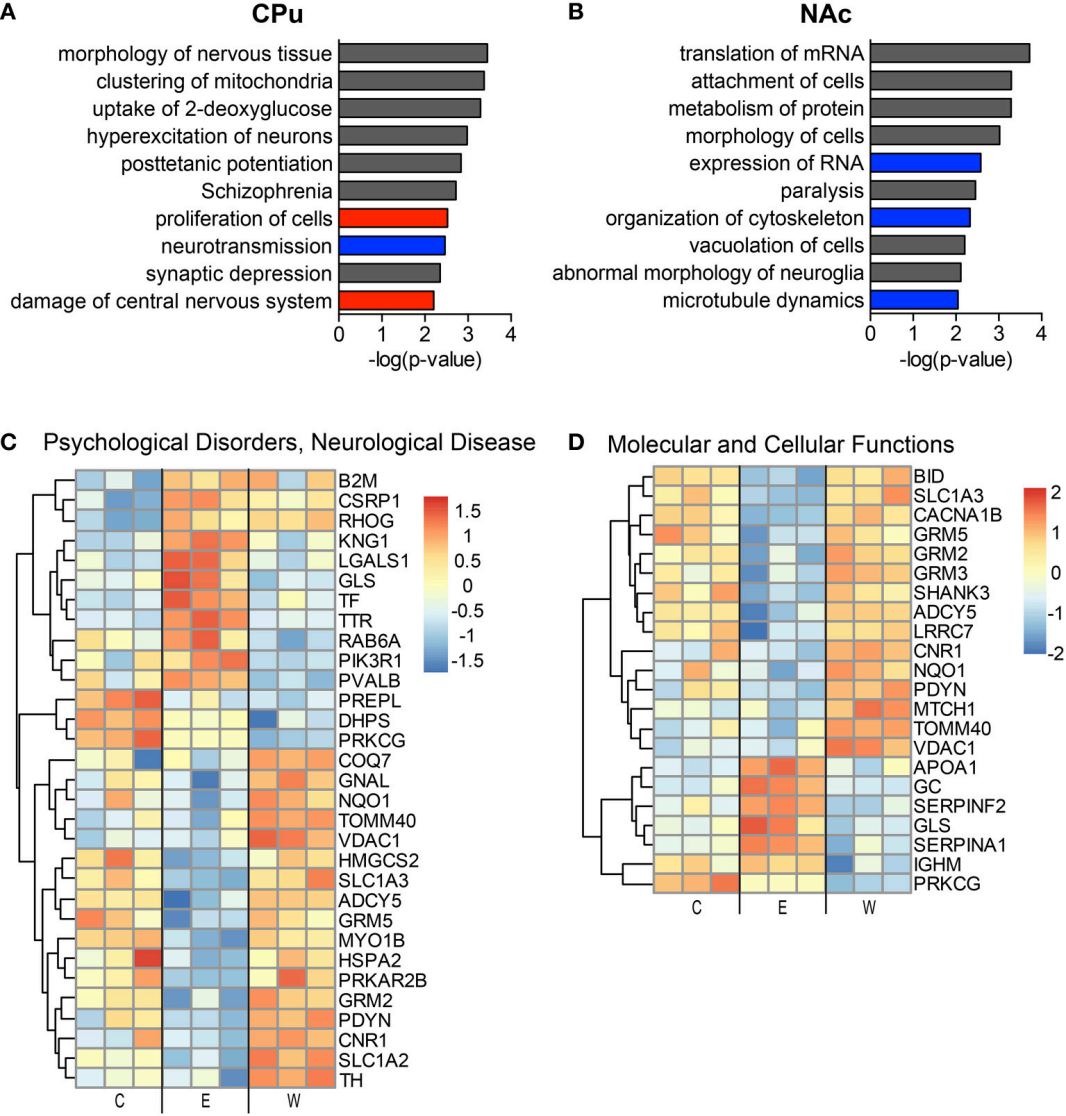
**The most significantly altered diseases and biological functions in the striatum of ethanol mice**. Up-regulated (red), down-regulated (blue), and indeterminable (gray) activation of diseases and biological functions in the caudate-putamen **(A)** and nucleus accumbens **(B)** are displayed according to overall significance (*p*-value). Normalized signal intensities of proteins within the network heat maps shown in **(C,D)** are from the CPu samples (inclusive of serum proteins). Complete data can be found in Supplemental Materials.

**Figure 7:**
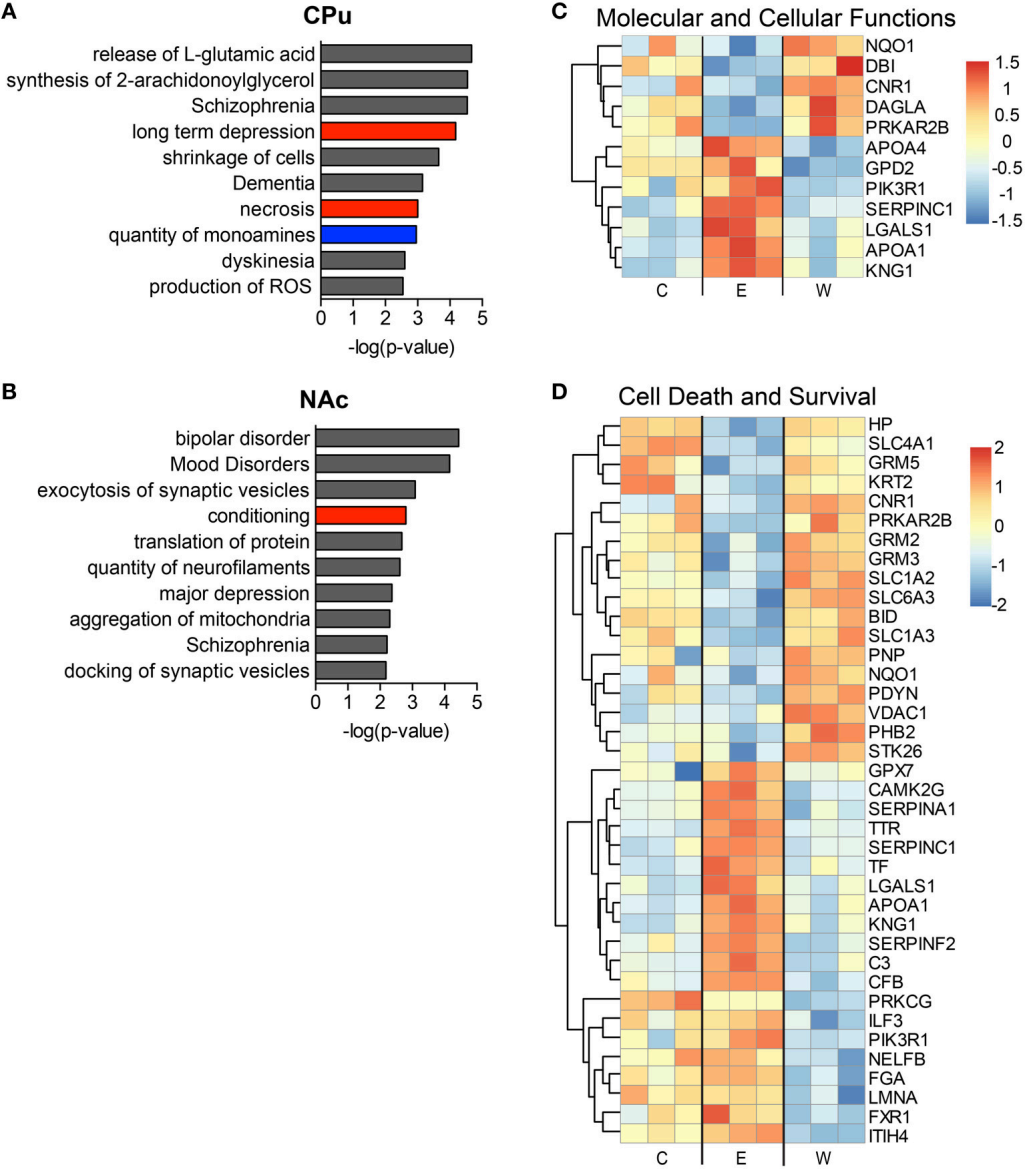
**The most significantly altered diseases and biological functions in the striatum of withdrawal mice**. Up-regulated (red), down-regulated (blue), and indeterminable (gray) activation of diseases and biological functions in the caudate-putamen **(A)** and nucleus accumbens **(B)** are displayed according to overall significance (*p*-value). Normalized signal intensities of proteins within the network heat maps shown in **(C,D)** are from the CPu samples (inclusive of serum proteins). Complete data can be found in Supplemental Materials.

The top implicated diseases and biological functions reflect the activated networks for the two conditions, with greater significance in overlap of molecules involved in molecular and cellular functions (Figure 6C) and cell death and survival (Figure 6D). In E mice (Figure 6), the biological functions of cell morphology (CPu; Figure 6A) and mRNA translation and cell attachment (NAc; Figure 6B) were the most significant, with diseases such as schizophrenia ranking less significant (CPu, *p*-value = 1.90E-03). However, more psychological disorders and neurological diseases (Figure 7; heat maps of pathway molecules in Figures 7C,D) were identified from the differentially altered proteins in W mice. Among these, schizophrenia (CPu; Figure 7A), and bipolar and mood disorders (NAc; Figure 7B) were the top diseases identified, along with biological functions of glutamate release and synaptic vesicle exocytosis (Figures 7A,B).

## Discussion

In this report we undertook a label-free proteomic approach to identify novel proteins associated with chronic ethanol exposure and more importantly, withdrawal from that condition. Unfortunately, a critical hallmark of alcohol addiction is the high occurrence of relapse following a period of abstinence. Therefore, it is of paramount importance that we elucidate novel proteins and their associated molecular pathways (affected by CIE and withdrawal) in order to adequately utilize pharmacological treatment strategies to help individuals suffering from this disease.

To model chronic alcohol use leading to dependence and subsequent withdrawal we utilized the CIE protocol, which has been demonstrated to accomplish these goals in previous studies (Becker et al., 1997; Becker and Lopez, 2004). After four cycles of CIE we were able to reproduce these previous findings, generating appropriate BACs to result in alcohol dependence and tolerance, validated by the increased alcohol consumption and shorter time to recovery from LORR by the mice that underwent the CIE procedure in comparison to controls.

Label-free proteomic analysis using LC-MS/MS combined with Ingenuity Pathway Analysis (IPA) revealed a plethora of altered proteins and activated pathways and networks. In order to focus on neuronal signaling pathways, we excluded many proteins primarily found in blood serum. We acknowledge that this may have resulted in reduced recognition of immune signaling and lipid/cholesterol pathways, and that validation and functional studies are warranted. Indeed, when these molecules were included in the IPA analysis (data not shown), we found increased significance of overlap with molecules involved in lipid/cholesterol (RXR signaling) pathways and acute phase response with molecules in our datasets.

Neuroproteomic analysis of the mouse striatum under conditions of CIE or acute withdrawal from CIE validated many previously identified signaling changes found in chronic ethanol and withdrawal treatments. For example, we found increased PDYN in withdrawal (Osterndorff-Kahanek et al., 2013), decreased CNR1 during chronic ethanol and increased LTD during withdrawal (DePoy et al., 2013), increased DRD3 (Rothblat et al., 2001) and decreased CAMKII signaling during withdrawal (Spiga et al., 2014). Additionally, top activated diseases and biological functions support recent theories and observations on the networks associated with alcohol withdrawal symptoms. For instance, medium spiny neurons in the putamen of animals after 4 weeks of CIE displayed increased neuronal excitability, and disruption of the inhibitory striatonigral circuit led to more severe withdrawal symptoms (Cuzon Carlson et al., 2011), reflecting the increases in synaptic transmission and increased glutamatergic signaling revealed in our withdrawal samples (see Figure 7A). Brain stress hormones also appear to affect the depression and anxiety symptoms seen during alcohol withdrawal (Meinhardt and Sommer, 2015), disease states that were implicated in the NAc dataset of our withdrawal samples (see Figure 7B). Also, anti-hypertensive medications that target the α- and β-adrenergic receptors, signaling pathways that were differentially expressed in our datasets, have been shown to improve withdrawal symptoms both clinically and in animals (Trzaskowska and Kostowski, 1983), supporting theories that the effects of these medications may be directly on neural circuitry and not simply through systemic actions.

Overall, we found more dramatic changes in protein levels of the CPu samples than we did with the NAc samples. One of the most striking findings was the significant increase in glutamate signaling in the CPu during ethanol withdrawal. This supports previous data that show increased glutamatergic neurotransmission during ethanol withdrawal (Tsai et al., 1998; Chen et al., 2011; Abulseoud et al., 2014). Increased excitatory signaling due to excess synaptic glutamate during withdrawal is considered to be a major contributing factor to alcohol withdrawal syndrome, thus stimulating discovery and testing of compounds potentially used to ease withdrawal symptoms (Bayard et al., 2004). For example, compounds like ceftriaxone show promising results (Rothstein et al., 2005; Rao et al., 2015), as it has been shown to increase the expression of glutamate transporters that remove excess synaptic glutamate. In support, our CPu neuroproteomic results confirm this increased glutamatergic signaling in the dorsal striatum, which may lead to dis-inhibition of the thalamus through both the direct and indirect pathways (Chen et al., 2011; Cuzon Carlson et al., 2011), as well as alterations in synaptic plasticity (Zhou and Danbolt, 2013).

Interestingly, we found that L-dopa, a catecholamine precursor, was identified as an increased upregulator in the CPu of W mice. Dopamine signaling, however, is decreased during withdrawal after chronic alcohol exposure (Berke and Hyman, 2000; Koob and Volkow, 2010; Karkhanis et al., 2015), though alcohol's effects on dopamine signaling are still somewhat controversial (Didone et al., 2016). Additionally, dopamine receptor and transporter upregulation has also been found under similar conditions (Rothblat et al., 2001), which may explain our findings. The involvement of the dopamine system in withdrawal symptoms has also been under investigation, as striatopallidal projections have been implicated in withdrawal tremors (Deik et al., 2012; Spiga et al., 2014), which appears to involve dopamine signaling. Dopamine signaling also seems to be involved in other psychotic symptoms (hallucinations, dementia) associated with AWS and schizophrenia (Rao et al., 2012). Notably, we found a significant overlap of pathway molecules associated with dementia in the NAc of our W mice.

Oxidative stress is a well-known cellular response to ethanol exposure (Tsai et al., 1998; Toth et al., 2010; Pochareddy and Edenberg, 2012; Moon et al., 2014), and local mitochondrial function has been shown to have an impact on dendritic morphogenesis and synaptic plasticity (Li et al., 2004). Many proteins involved in oxidative stress and the mitochondrial electron transport chain were affected in our chronic ethanol and acute withdrawal samples, although mainly components of complex I were significantly increased during withdrawal. The NADH dehydrogenase complex I has been implicated in certain neuropsychiatric disorders, such as bipolar disorder and major depressive disorder (Andreazza et al., 2010; Mazereeuw et al., 2015), as its dysfunction results in increased ROS leading to a multitude of adverse consequences (Trivedi and Deth, 2014), including activation of the NLRP3 inflammasome and lipid peroxidation. Incidentally, several pathways involved in lipid/cholesterol metabolism and inflammatory responses (LXR/RXR, FXR/RXR, acute phase response) were differentially activated in the E and W mice (details can be found in Supplemental Data sheets 2, 3). Alterations in plasma membrane lipid composition can affect surface protein trafficking and function (Tobin et al., 2014), such as glutamate transport by excitatory amino acid transporters (EAAT's) (Butchbach et al., 2004). Perhaps plasma membrane composition and integrity play a greater role in the functions of key receptors and transporters in the striatum than was previously thought. Also, the inflammatory response to alcohol and its withdrawal is receiving increasing attention, as astrocytes and microglia have been shown to play a key role in neuronal integrity and synaptic plasticity, both of which are affected in AUD (Crews et al., 2013; Olmos and Lladó, 2014).

Though much of our data confirm established consequences of chronic ethanol use and withdrawal, our results support the need for increased research into the application and development of therapeutics targeted toward mitochondrial dysfunction and reduction of glutamate transmission (possibly through increased glutamate transport). Additionally, utilization of substances improving plasma membrane integrity may be another avenue to consider when treating AWS. We hope that these proteomic analyses will prove useful for others investigating the causes of AWS and treatment methods to aid in recovery from AUD.

## Author contributions

JA AO, DL, DH, and DC wrote manuscript. JA, AO, and DC designed research. JA, YQ, and AO performed research. JA, AO, RM, SD, and DC analyzed data. All authors have read the manuscript and approve the final manuscript.

## Funding

This work was supported by the Samuel C. Johnson for Genomics of Addiction Program at Mayo Clinic, the Ulm Foundation, the Godby Foundation, David Lehr Research Award from American Society for Pharmacology and Experimental Therapeutics, Mayo-Karolinska Institute (KI) Research Award and National Institute on Alcohol Abuse and Alcoholism (AA018779).

### Conflict of interest statement

The authors declare that the research was conducted in the absence of any commercial or financial relationships that could be construed as a potential conflict of interest.
