# Supplementary material for: Label-Free Proteomic Analysis of Protein Changes in the Striatum during Chronic Ethanol Use and Early Withdrawal
Source: Front Behav Neurosci. 2016 Mar 11;10:46. doi: 10.3389/fnbeh.2016.00046 (PMC4786553; doi:10.3389/fnbeh.2016.00046)
Supplement: Supplementary file 1 [file Table1.DOCX]

| **E vs C** | | **CPu** |
| --- | --- | --- |
| Score | Focus Molecules | Top Diseases and Functions |
| 21 | 15 | Cell Morphology, Cellular Development, Cellular Growth and Proliferation |
| 17 | 13 | Cell-To-Cell Signaling and Interaction, Nervous System Development and Function, Neurological Disease |
| 15 | 12 | Cardiovascular System Development and Function, Organismal Development, Cellular Movement |
| 13 | 11 | Neurological Disease, Skeletal and Muscular Disorders, Psychological Disorders |
| 6 | 5 | Cellular Growth and Proliferation, Gene Expression, Cell Cycle |
|  | | **NAc** |
| 19 | 15 | Tissue Development, Cell Death and Survival, Cardiovascular System Development and Function |
| 18 | 14 | Embryonic Development, Organismal Development, Cell Morphology |
| 18 | 14 | Neurological Disease, Cellular Development, Nervous System Development and Function |
| 16 | 13 | Free Radical Scavenging, Cardiovascular System Development and Function, Hematological System Development and Function |
| 12 | 11 | Cellular Development, Cellular Growth and Proliferation, Nervous System Development and Function |

**Table S1**. Top activated networks in the striatum of mice undergoing chronic intermittent ethanol treatments (relative to air controls)
