# Supplementary material for: Label-Free Proteomic Analysis of Protein Changes in the Striatum during Chronic Ethanol Use and Early Withdrawal
Source: Front Behav Neurosci. 2016 Mar 11;10:46. doi: 10.3389/fnbeh.2016.00046 (PMC4786553; doi:10.3389/fnbeh.2016.00046)
Supplement: Supplementary file 2 [file Table2.DOCX]

**Table S2**. Top activated networks in the striatum of mice during acute withdrawal from chronic intermittent ethanol (CIE) treatments (relative to CIE mice). *Different proteins were altered in sub-categories of the “Developmental Disorder, Hereditary Disorder, Metabolic Disease” network in the NAc.*

| **W vs E** | | **CPu** |
| --- | --- | --- |
| Score | Focus Molecules | Top Diseases and Functions |
| 24 | 17 | Behavior, Cell-To-Cell Signaling and Interaction, Drug Metabolism |
| 24 | 17 | Cell Death and Survival, Behavior, Lipid Metabolism |
| 18 | 14 | Neurological Disease, Psychological Disorders, Skeletal and Muscular Disorders |
| 18 | 14 | Cell Death and Survival, Cellular Assembly and Organization, Cellular Development |
| 15 | 12 | Behavior, Neurological Disease, Psychological Disorders |
|  | | **NAc** |
| 20 | 15 | Biliary Hyperplasia, Hepatic System Development and Function, Liver Cholestasis |
| 17 | 15 | Cell-To-Cell Signaling and Interaction, Nervous System Development and Function, Amino Acid Metabolism |
| 17 | 15 | Molecular Transport, Small Molecule Biochemistry, Cell-To-Cell Signaling and Interaction |
| 15 | 14 | Behavior, Nervous System Development and Function, Neurological Disease |
| 15 | 14 | Behavior, Cell-To-Cell Signaling and Interaction, Nervous System Development and Function |
