# Supplementary material for: Label-Free Proteomic Analysis of Protein Changes in the Striatum during Chronic Ethanol Use and Early Withdrawal
Source: Front Behav Neurosci. 2016 Mar 11;10:46. doi: 10.3389/fnbeh.2016.00046 (PMC4786553; doi:10.3389/fnbeh.2016.00046)
Supplement: Supplementary file 3 [file DataSheet1.PDF]

INGENUITY®
PATHWAY ANALYSIS

Analysis Name: CPu-EvC no serum-neuro restrict
Analysis Creation Date: 2015-11-19
Build version: 355958M
Content version: 24718999 (Release Date: 2015-09-14)

Analysis Settings

Reference set: Ingenuity Knowledge Base (Genes Only)

Relationship to include: Direct and Indirect

Includes Endogenous Chemicals

Optional Analyses: My Pathways My List

Filter Summary:

Consider only molecules and/or relationships where

(confidence = Experimentally Observed) AND

(tissues = Stem cells not otherwise specified OR Dorsal Root Ganglion OR Gray Matter OR White Matter OR Sciatic Nerve OR Parietal Lobe OR Neurons not otherwise specified OR Cortical neurons OR Cerebral Ventricles OR Thalamus OR Subventricular Zone OR Corpus Callosum OR Nervous System not otherwise specified OR Amygdala OR Trigeminal Ganglion OR Brain OR Hippocampus OR Microvascular endothelial cells OR Granule cells OR Brainstem OR Purkinje cells OR Pituitary Gland OR Other Neurons OR Stromal cells OR Endothelial cells not otherwise specified OR Nucleus Accumbens OR Olfactory Bulb OR Astrocytes OR Other Nervous System OR Choroid Plexus OR Microglia OR Striatum OR Ventricular Zone OR Hypothalamus OR Substantia Nigra OR Spinal Cord OR Cells not otherwise specified OR Caudate Nucleus OR Cerebral Cortex OR Cerebellum OR Adipocytes OR Medulla Oblongata OR Pyramidal neurons OR Granule Cell Layer OR Putamen OR Other Stem cells)

(c) 2000-2015 QIAGEN. All rights reserved.

INGENUITY® SYSTEMS

Top Canonical Pathways

Name	p-value	Overlap
Synaptic Long Term Potentiation	2.26E-05	6.9 % 7/101
Protein Kinase A Signaling	3.84E-05	3.7 % 11/295
Melatonin Signaling	1.48E-04	8.3 % 5/60
Dopamine-DARPP32 Feedback in cAMP Signaling	1.65E-04	5.1 % 7/138
GPCR-Mediated Nutrient Sensing in Enteroendocrine Cells	3.07E-04	7.1 % 5/70

Top Upstream Regulators

Upstream Regulator	p-value of overlap	Predicted Activation
RICTOR	1.08E-05	
L-glutamic acid	1.25E-03	
JAK2	1.84E-03	
HTT	2.08E-03	
PPP3CA	3.22E-03	

Top Diseases and Bio Functions

Diseases and Disorders

Name	p-value	#Molecules
Neurological Disease	4.16E-02 - 1.90E-03	18
Psychological Disorders	1.68E-02 - 1.90E-03	14
Hereditary Disorder	1.68E-02 - 4.03E-03	8
Skeletal and Muscular Disorders	8.20E-03 - 4.03E-03	9
Cancer	8.46E-03 - 8.46E-03	1

Molecular and Cellular Functions

(c) 2000-2015 QIAGEN. All rights reserved.

Name	p-value	#Molecules
Cellular Assembly and Organization	4.97E-02 - 4.20E-04	14
Carbohydrate Metabolism	5.16E-04 - 5.16E-04	3
Molecular Transport	1.68E-02 - 5.16E-04	5
Small Molecule Biochemistry	3.52E-02 - 5.16E-04	8
Cell Morphology	4.97E-02 - 8.75E-04	22

Physiological System Development and Function

Name	p-value	#Molecules
Nervous System Development and Function	4.97E-02 - 3.54E-04	37
Tissue Morphology	4.97E-02 - 3.54E-04	17
Organ Morphology	4.97E-02 - 1.04E-03	13
Organismal Development	4.97E-02 - 1.04E-03	20
Endocrine System Development and Function	4.97E-02 - 8.46E-03	3

Top Networks

ID	Associated Network Functions	Score
1	Cell Morphology, Cellular Development, Cellular Growth and Proliferation	21
2	Cell-To-Cell Signaling and Interaction, Nervous System Development and Function, Neurological Disease	17
3	Cardiovascular System Development and Function, Organismal Development, Cellular Movement	15
4	Neurological Disease, Skeletal and Muscular Disorders, Psychological Disorders	13
5	Cellular Growth and Proliferation, Gene Expression, Cell Cycle	6

Top Tox Lists

Name	p-value	Overlap
Negative Acute Phase Response Proteins	6.97E-04	40.0 % 2/5
RAR Activation	2.65E-03	3.7 % 6/164

(c) 2000-2015 QIAGEN. All rights reserved.

INGENUITY® SYSTEMS

Mechanism of Gene Regulation by Peroxisome Proliferators via PPAR	6.53E-03	4.5 % 4/88
Hepatic Cholestasis	6.85E-03	3.5 % 5/141
PPAR/RXR Activation	9.32E-03	3.3 % 5/152

Top My Lists

Name	p-value	Overlap
66 EvC only (mostly neuronal) molecules	5.74E-109	98.0 % 50/51

Top Analysis-Ready Molecules

Exp Fold Change up-regulated

Molecules	Exp. Value	Exp. Chart
**TRIM32**	↑ 43443686634.987	
**TLE4**	↑ 10296483346.115	
LIAS	↑ 6.968	
**SERPINC1**	↑ 5.965	
**GPX3**	↑ 4.745	
**TTR**	↑ 4.582	
MICALL1	↑ 4.460	
**TP53I11**	↑ 2.898	
**SERPINF2**	↑ 2.839	
PLEKHB1	↑ 2.653	

Exp Fold Change down-regulated

Molecules	Exp. Value	Exp. Chart
**BID**	↓ -7.634	
**HAP1**	↓ -5.385	
**HP**	↓ -3.997	
PTPDC1	↓ -3.799	

(c) 2000-2015 QIAGEN. All rights reserved.

INGENUITY SYSTEMS

**PLXNC1**	↓ -3.310
**MYO1B**	↓ -3.130
**AKAP2**	↓ -2.939
EML6	↓ -2.678
**RGS14**	↓ -2.647
WFS1	↓ -2.482

(c) 2000-2015 QIAGEN. All rights reserved.

INGENUITY SYSTEMS

INGENUITY
PATHWAY ANALYSIS

Analysis Name: CPu-WvE no serum-neuro restrict
Analysis Creation Date: 2015-11-19
Build version: 355958M
Content version: 24718999 (Release Date: 2015-09-14)

Analysis Settings

Reference set: Ingenuity Knowledge Base (Genes Only)
Relationship to include: Direct and Indirect
Includes Endogenous Chemicals
Optional Analyses: My Pathways My List

Filter Summary:
Consider only molecules and/or relationships where
(confidence = Experimentally Observed) AND
(tissues = Stem cells not otherwise specified OR Dorsal Root Ganglion OR Gray Matter OR White Matter OR Sciatic Nerve OR Parietal Lobe OR Neurons not otherwise specified OR Cortical neurons OR Cerebral Ventricles OR Thalamus OR Subventricular Zone OR Corpus Callosum OR Nervous System not otherwise specified OR Amygdala OR Trigeminal Ganglion OR Brain OR Hippocampus OR Microvascular endothelial cells OR Granule cells OR Brainstem OR Purkinje cells OR Pituitary Gland OR Other Neurons OR Stromal cells OR Endothelial cells not otherwise specified OR Nucleus Accumbens OR Olfactory Bulb OR Astrocytes OR Other Nervous System OR Choroid Plexus OR Microglia OR Striatum OR Ventricular Zone OR Hypothalamus OR Substantia Nigra OR Spinal Cord OR Cells not otherwise specified OR Caudate Nucleus OR Cerebral Cortex OR Cerebellum OR Adipocytes OR Medulla Oblongata OR Pyramidal neurons OR Granule Cell Layer OR Putamen OR Other Stem cells)

(c) 2000-2015 QIAGEN. All rights reserved.

INGENUITY SYSTEMS

Top Canonical Pathways

Name	p-value	Overlap	
CREB Signaling in Neurons	1.35E-05	6.0 %	9/151
Glutamate Receptor Signaling	1.61E-05	10.5 %	6/57
Neuropathic Pain Signaling In Dorsal Horn Neurons	1.86E-05	8.0 %	7/87
G-Protein Coupled Receptor Signaling	8.71E-05	4.2 %	10/237
Synaptic Long Term Potentiation	4.00E-04	5.9 %	6/101

Top Upstream Regulators

Upstream Regulator	p-value of overlap	Predicted Activation
GRM3	3.04E-06	
HTT	5.26E-06	
L-dopa	7.05E-05	Activated
DRD3	2.51E-04	
NR4A2	4.73E-04	

Top Diseases and Bio Functions

Diseases and Disorders

Name	p-value	#Molecules
Neurological Disease	4.68E-02 - 2.90E-05	30
Psychological Disorders	4.68E-02 - 2.90E-05	26
Metabolic Disease	9.54E-03 - 2.77E-03	8
Hereditary Disorder	3.76E-02 - 8.25E-03	9
Skeletal and Muscular Disorders	3.76E-02 - 8.25E-03	9

Molecular and Cellular Functions

(c) 2000-2015 QIAGEN. All rights reserved.

INGENUITY SYSTEMS

Name	p-value	#Molecules
Amino Acid Metabolism	2.83E-02 - 2.15E-05	8
Cell-To-Cell Signaling and Interaction	4.68E-02 - 2.15E-05	20
Molecular Transport	4.68E-02 - 2.15E-05	14
Small Molecule Biochemistry	4.68E-02 - 2.15E-05	15
Lipid Metabolism	2.00E-02 - 2.88E-05	7

Physiological System Development and Function

Name	p-value	#Molecules
Endocrine System Development and Function	4.68E-02 - 2.88E-05	4
Nervous System Development and Function	4.68E-02 - 6.68E-05	21
Embryonic Development	2.83E-02 - 8.85E-04	7
Organ Development	4.68E-02 - 8.85E-04	3
Organismal Development	9.54E-03 - 8.85E-04	7

Top Networks

ID	Associated Network Functions	Score
1	Behavior, Cell-To-Cell Signaling and Interaction, Drug Metabolism	24
2	Cell Death and Survival, Behavior, Lipid Metabolism	24
3	Neurological Disease, Psychological Disorders, Skeletal and Muscular Disorders	18
4	Cell Death and Survival, Cellular Assembly and Organization, Cellular Development	18
5	Behavior, Neurological Disease, Psychological Disorders	15

Top Tox Lists

Name	p-value	Overlap
FXR/RXR Activation	8.78E-03	4.7 % 4/85
Mitochondrial Dysfunction	1.03E-02	3.6 % 5/138

(c) 2000-2015 QIAGEN. All rights reserved.

Positive Acute Phase Response Proteins	1.39E-02	10.5 % 2/19
PXR/RXR Activation	1.40E-02	5.7 % 3/53
Hypoxia-Inducible Factor Signaling	2.04E-02	4.9 % 3/61

Top My Lists

Name	p-value	Overlap
90 WvE only (mostly neuronal) molecules	1.53E-132	92.8 % 64/69

Top Analysis-Ready Molecules

Exp Fold Change up-regulated

Molecules	Exp. Value	Exp. Chart
**PDYN**	↑ 8.682	
**BID**	↑ 8.642	
TMSB10/TMSB4X	↑ 4.609	
**CNR1**	↑ 2.783	
COQ7	↑ 2.745	
**MYO1B**	↑ 2.635	
**SLC6A3**	↑ 2.497	
**SLC1A3**	↑ 2.370	
STK26	↑ 2.325	
WFS1	↑ 2.252	

Exp Fold Change down-regulated

Molecules	Exp. Value	Exp. Chart
LIAS	↓ -7.721	
RPL28	↓ -6.233	
CHERP	↓ -6.140	
**SERPINC1**	↓ -6.102	

(c) 2000-2015 QIAGEN. All rights reserved.

**SERPINF2**	↓ -4.441
KIAA1217	↓ -4.407
**ILF3**	↓ -4.204
**TTR**	↓ -4.120
PJA2	↓ -3.574
**GPX3**	↓ -3.330

(c) 2000-2015 QIAGEN. All rights reserved.

INGENUITY® SYSTEMS

INGENUITY
PATHWAY ANALYSIS

Analysis Name: CPu-WvC (NS,NR)
Analysis Creation Date: 2015-11-27
Build version: 355958M
Content version: 24718999 (Release Date: 2015-09-14)

Analysis Settings

Reference set: Ingenuity Knowledge Base (Genes Only)

Relationship to include: Direct and Indirect

Includes Endogenous Chemicals

Optional Analyses: My Pathways My List

Filter Summary:

Consider only molecules and/or relationships where

(confidence = Experimentally Observed) AND

(tissues = Amygdala OR Cells not otherwise specified OR Cerebellum OR Brainstem OR Hippocampus OR Medulla Oblongata OR Granule Cell Layer OR Granule cells OR Pituitary Gland OR Microvascular endothelial cells OR Stromal cells OR Other Neurons OR Gray Matter OR Cortical neurons OR Other Nervous System OR Astrocytes OR Parietal Lobe OR Neurons not otherwise specified OR Olfactory Bulb OR Striatum OR Cerebral Ventricles OR Corpus Callosum OR Substantia Nigra OR Thalamus OR Caudate Nucleus OR Trigeminal Ganglion OR Brain OR Nervous System not otherwise specified OR Cerebral Cortex OR Putamen OR Sciatic Nerve OR Purkinje cells OR Adipocytes OR Pyramidal neurons OR Other Stem cells OR White Matter OR Dorsal Root Ganglion OR Endothelial cells not otherwise specified OR Nucleus Accumbens OR Choroid Plexus OR Microglia OR Subventricular Zone OR Ventricular Zone OR Hypothalamus OR Stem cells not otherwise specified OR Spinal Cord)

(c) 2000-2015 QIAGEN. All rights reserved.

INGENUITY SYSTEMS

Top Canonical Pathways

Name	p-value	Overlap	
Mitochondrial Dysfunction	5.42E-08	10.1 %	14/138
Oxidative Phosphorylation	1.10E-07	13.1 %	11/84
VEGF Signaling	2.35E-04	9.2 %	7/76
Cholecystokinin/Gastrin-mediated Signaling	7.09E-04	7.7 %	7/91
Glycogen Biosynthesis II (from UDP-D-Glucose)	7.88E-04	66.7 %	2/3

Top Upstream Regulators

Upstream Regulator	p-value of overlap	Predicted Activation
RICTOR	7.67E-05	
BDNF	1.85E-04	
ROR1	2.52E-04	
ROR2	2.52E-04	
HTT	3.00E-04	

Top Diseases and Bio Functions

Diseases and Disorders

Name	p-value	#Molecules
Neurological Disease	4.95E-02 - 1.82E-05	39
Organismal Injury and Abnormalities	4.95E-02 - 5.80E-04	21
Psychological Disorders	4.82E-02 - 1.05E-03	27
Skeletal and Muscular Disorders	4.95E-02 - 1.22E-03	20
Inflammatory Response	4.82E-02 - 2.57E-03	5

Molecular and Cellular Functions

(c) 2000-2015 QIAGEN. All rights reserved.

Name	p-value	#Molecules
Cell Morphology	4.82E-02 - 2.13E-06	49
Cellular Assembly and Organization	4.82E-02 - 3.98E-06	43
Cellular Function and Maintenance	4.82E-02 - 3.98E-06	40
Cellular Development	4.82E-02 - 7.26E-06	46
Cell-To-Cell Signaling and Interaction	4.95E-02 - 1.14E-04	31

Physiological System Development and Function

Name	p-value	#Molecules
Nervous System Development and Function	4.95E-02 - 5.21E-06	66
Tissue Morphology	4.12E-02 - 5.21E-06	41
Tissue Development	4.82E-02 - 7.26E-06	42
Organ Morphology	4.82E-02 - 1.09E-05	27
Organismal Development	4.82E-02 - 1.09E-05	33

Top Tox Functions

Assays: Clinical Chemistry and Hematology

Name	p-value	#Molecules
Decreased Levels of Albumin	1.63E-02 - 1.63E-02	1

Cardiotoxicity

Name	p-value	#Molecules
Pulmonary Hypertension	1.63E-02 - 1.63E-02	1

(c) 2000-2015 QIAGEN. All rights reserved.

INGENUITY® SYSTEMS

Top Networks

ID	Associated Network Functions	Score
1	Behavior, Cellular Development, Cellular Growth and Proliferation	16
2	Cell Death and Survival, Cellular Development, Nervous System Development and Function	15
3	Neurological Disease, Cell Death and Survival, Psychological Disorders	13
4	Cell Death and Survival, Cancer, Organismal Injury and Abnormalities	13
5	Behavior, Cell Death and Survival, Cellular Movement	13

Top Tox Lists

Name	p-value	Overlap	
Mitochondrial Dysfunction	5.42E-08	10.1 %	14/138
Hypoxia-Inducible Factor Signaling	3.14E-03	8.2 %	5/61
RAR Activation	5.49E-03	4.9 %	8/164
Cardiac Fibrosis	2.16E-02	4.1 %	7/170
VDR/RXR Activation	3.28E-02	5.4 %	4/74

Top My Lists

Name	p-value	Overlap	
175 WvC only (mostly neuronal) molecules	2.28E-299	99.3 %	149/150

Top Analysis-Ready Molecules

Exp Fold Change up-regulated

Molecules	Exp. Value	Exp. Chart
MICALL1	↑ 4.020	
**TRPV2**	↑ 3.506	
**ENDOG**	↑ 2.890	
**NDUFS6**	↑ 2.726	
MZT2A	↑ 2.580	

(c) 2000-2015 QIAGEN. All rights reserved.

**COX6A1**	↑ 2.387
**CAV1**	↑ 2.282
TIMM22	↑ 2.269
**CLNS1A**	↑ 2.259
**MOBP**	↑ 2.173

Exp Fold Change down-regulated

Molecules	Exp. Value	Exp. Chart
**HOMER2**	↓ -35054557201.723	
**FHL2**	↓ -4588652106.352	
BAIAP3	↓ -4.829	
**NGB**	↓ -4.607	
GYG1	↓ -3.816	
PJA2	↓ -3.573	
DHPS	↓ -3.042	
RPL18	↓ -2.856	
**ATF2**	↓ -2.807	
**AHI1**	↓ -2.652	

(c) 2000-2015 QIAGEN. All rights reserved.
