# Supplementary material for: Label-Free Proteomic Analysis of Protein Changes in the Striatum during Chronic Ethanol Use and Early Withdrawal
Source: Front Behav Neurosci. 2016 Mar 11;10:46. doi: 10.3389/fnbeh.2016.00046 (PMC4786553; doi:10.3389/fnbeh.2016.00046)
Supplement: Supplementary file 10 [file DataSheet8.PDF]

INGENUITY
PATHWAY ANALYSIS

Analysis Name: NAc-EvC (triplicates) no serum-neuro restrict
Analysis Creation Date: 2015-11-19
Build version: 355958M
Content version: 24718999 (Release Date: 2015-09-14)

Analysis Settings

Reference set: Ingenuity Knowledge Base (Genes Only)

Relationship to include: Direct and Indirect

Includes Endogenous Chemicals

Optional Analyses: My Pathways My List

Filter Summary:

Consider only molecules and/or relationships where

(confidence = Experimentally Observed) AND

(tissues = Stem cells not otherwise specified OR Dorsal Root Ganglion OR Gray Matter OR White Matter OR Sciatic Nerve OR Parietal Lobe OR Neurons not otherwise specified OR Cortical neurons OR Cerebral Ventricles OR Thalamus OR Subventricular Zone OR Corpus Callosum OR Nervous System not otherwise specified OR Amygdala OR Trigeminal Ganglion OR Brain OR Hippocampus OR Microvascular endothelial cells OR Granule cells OR Brainstem OR Purkinje cells OR Pituitary Gland OR Other Neurons OR Stromal cells OR Endothelial cells not otherwise specified OR Nucleus Accumbens OR Olfactory Bulb OR Astrocytes OR Other Nervous System OR Choroid Plexus OR Microglia OR Striatum OR Ventricular Zone OR Hypothalamus OR Substantia Nigra OR Spinal Cord OR Cells not otherwise specified OR Caudate Nucleus OR Cerebral Cortex OR Cerebellum OR Adipocytes OR Medulla Oblongata OR Pyramidal neurons OR Granule Cell Layer OR Putamen OR Other Stem cells)

(c) 2000-2015 QIAGEN. All rights reserved.

INGENUITY SYSTEMS

Top Canonical Pathways

Name	p-value	Overlap	
EIF2 Signaling	7.81E-10	9.8 %	13/133
mTOR Signaling	9.49E-04	4.5 %	7/154
Regulation of eIF4 and p70S6K Signaling	1.09E-03	5.2 %	6/115
Pentose Phosphate Pathway	2.73E-03	25.0 %	2/8
Histamine Degradation	5.26E-03	18.2 %	2/11

Top Upstream Regulators

Upstream Regulator	p-value of overlap	Predicted Activation
MYC	1.63E-06	
CPLX1	9.93E-03	
CPLX2	9.93E-03	
GABRD	9.93E-03	
MTTP	9.93E-03	

Top Diseases and Bio Functions

Diseases and Disorders

Name	p-value	#Molecules
Neurological Disease	4.96E-02 - 3.49E-03	8
Cancer	3.00E-02 - 1.01E-02	2
Cardiovascular Disease	1.01E-02 - 1.01E-02	1
Gastrointestinal Disease	1.01E-02 - 1.01E-02	1
Hepatic System Disease	1.01E-02 - 1.01E-02	1

Molecular and Cellular Functions

(c) 2000-2015 QIAGEN. All rights reserved.

INGENUITY® SYSTEMS

Name	p-value	#Molecules
Gene Expression	3.00E-02 - 1.90E-04	10
Protein Synthesis	1.01E-02 - 1.90E-04	8
Cell-To-Cell Signaling and Interaction	3.99E-02 - 3.02E-04	11
Cell Morphology	4.96E-02 - 9.53E-04	30
Small Molecule Biochemistry	4.96E-02 - 2.06E-03	7

Physiological System Development and Function

Name	p-value	#Molecules
Nervous System Development and Function	4.96E-02 - 2.06E-03	29
Tissue Development	4.13E-02 - 3.36E-03	21
Cardiovascular System Development and Function	2.01E-02 - 8.53E-03	5
Organismal Development	4.13E-02 - 8.53E-03	20
Tissue Morphology	4.96E-02 - 9.11E-03	17

Top Tox Functions

Assays: Clinical Chemistry and Hematology

Name	p-value	#Molecules
Decreased Levels of Potassium	1.01E-02 - 1.01E-02	1

Top Networks

ID	Associated Network Functions	Score
1	Tissue Development, Cell Death and Survival, Cardiovascular System Development and Function	19
2	Embryonic Development, Organismal Development, Cell Morphology	18
3	Neurological Disease, Cellular Development, Nervous System Development and Function	18

(c) 2000-2015 QIAGEN. All rights reserved.

INGENUITY® SYSTEMS

4	Free Radical Scavenging, Cardiovascular System Development and Function, Hematological System Development and Function	16
5	Cellular Development, Cellular Growth and Proliferation, Nervous System Development and Function	12

Top My Lists

Name	p-value	Overlap
66 EvC only (mostly neuronal) molecules	1.48E-02	5.9 % 3/51
90 WvE only (mostly neuronal) molecules	1.54E-01	2.9 % 2/69
175 WvC only (mostly neuronal) molecules	4.50E-01	1.3 % 2/150

Top Analysis-Ready Molecules

Exp Fold Change up-regulated

Molecules	Exp. Value	Exp. Chart
**CENPE**	↑ 30688328052.500	
PID1	↑ 18174517989.763	
**ITGA3**	↑ 5670637071.568	
H2AFY	↑ 6.684	
Commd6	↑ 4.898	
**PDLIM4**	↑ 4.041	
**CPLX3**	↑ 2.899	
**HSPG2**	↑ 2.867	
H3F3A/H3F3B	↑ 2.737	
**DNMT1**	↑ 2.664	

Exp Fold Change down-regulated

Molecules	Exp. Value	Exp. Chart
**ADD1***	↓ -5.359	
CLCN4	↓ -4.533	

(c) 2000-2015 QIAGEN. All rights reserved.

INGENUITY SYSTEMS

**KCNQ3**	↓ -4.013
GDPD5	↓ -3.503
ELFN1	↓ -3.385
**LAMP5**	↓ -3.080
GPCPD1	↓ -2.902
**MCTP1**	↓ -2.854
**SMAD2**	↓ -2.801
**KIRREL3**	↓ -2.746

(c) 2000-2015 QIAGEN. All rights reserved.

INGENUITY SYSTEMS

INGENUITY
PATHWAY ANALYSIS

Analysis Name: NAc-WvE (triplicates) no serum-neuro restrict
Analysis Creation Date: 2015-11-19
Build version: 355958M
Content version: 24718999 (Release Date: 2015-09-14)

Analysis Settings

Reference set: Ingenuity Knowledge Base (Genes Only)

Relationship to include: Direct and Indirect

Includes Endogenous Chemicals

Optional Analyses: My Pathways My List

Filter Summary:

Consider only molecules and/or relationships where

(confidence = Experimentally Observed) AND

(tissues = Stem cells not otherwise specified OR Dorsal Root Ganglion OR Gray Matter OR White Matter OR Sciatic Nerve OR Parietal Lobe OR Neurons not otherwise specified OR Cortical neurons OR Cerebral Ventricles OR Thalamus OR Subventricular Zone OR Corpus Callosum OR Nervous System not otherwise specified OR Amygdala OR Trigeminal Ganglion OR Brain OR Hippocampus OR Microvascular endothelial cells OR Granule cells OR Brainstem OR Purkinje cells OR Pituitary Gland OR Other Neurons OR Stromal cells OR Endothelial cells not otherwise specified OR Nucleus Accumbens OR Olfactory Bulb OR Astrocytes OR Other Nervous System OR Choroid Plexus OR Microglia OR Striatum OR Ventricular Zone OR Hypothalamus OR Substantia Nigra OR Spinal Cord OR Cells not otherwise specified OR Caudate Nucleus OR Cerebral Cortex OR Cerebellum OR Adipocytes OR Medulla Oblongata OR Pyramidal neurons OR Granule Cell Layer OR Putamen OR Other Stem cells)

(c) 2000-2015 QIAGEN. All rights reserved.

Top Canonical Pathways

Name	p-value	Overlap
Oxidative Phosphorylation	1.26E-22	27.4 % 23/84
Mitochondrial Dysfunction	9.76E-20	18.1 % 25/138
EIF2 Signaling	1.34E-16	16.5 % 22/133
Cardiac -adrenergic Signaling	5.86E-04	6.8 % 8/118
Regulation of eIF4 and p70S6K Signaling	2.40E-03	6.1 % 7/115

Top Upstream Regulators

Upstream Regulator	p-value of overlap	Predicted Activation
MYC	5.00E-08	Inhibited
HTT	1.58E-06	
MAPT	1.37E-03	
PSEN1	2.91E-03	
REST	3.99E-03	

Top Diseases and Bio Functions

Diseases and Disorders

Name	p-value	#Molecules
Neurological Disease	4.70E-02 - 3.68E-05	30
Psychological Disorders	3.16E-02 - 3.68E-05	16
Organismal Injury and Abnormalities	4.70E-02 - 2.52E-03	13
Cancer	4.70E-02 - 1.59E-02	2
Metabolic Disease	1.59E-02 - 1.59E-02	1

Molecular and Cellular Functions

(c) 2000-2015 QIAGEN. All rights reserved.

INGENUITY SYSTEMS

Name	p-value	#Molecules
Cellular Assembly and Organization	4.81E-02 - 7.49E-04	31
Cellular Development	4.81E-02 - 7.49E-04	24
Cellular Growth and Proliferation	4.81E-02 - 7.49E-04	14
Cellular Function and Maintenance	4.70E-02 - 8.20E-04	25
Cellular Movement	3.16E-02 - 8.20E-04	7

Physiological System Development and Function

Name	p-value	#Molecules
Nervous System Development and Function	5.00E-02 - 7.49E-04	30
Tissue Development	4.81E-02 - 7.49E-04	24
Behavior	1.59E-02 - 1.58E-03	4
Embryonic Development	4.70E-02 - 2.44E-03	12
Tissue Morphology	5.00E-02 - 2.44E-03	12

Top Networks

ID	Associated Network Functions	Score
1	Biliary Hyperplasia, Hepatic System Development and Function, Liver Cholestasis	20
2	Cell-To-Cell Signaling and Interaction, Nervous System Development and Function, Amino Acid Metabolism	17
3	Molecular Transport, Small Molecule Biochemistry, Cell-To-Cell Signaling and Interaction	17
4	Behavior, Nervous System Development and Function, Neurological Disease	15
5	Behavior, Cell-To-Cell Signaling and Interaction, Nervous System Development and Function	15

Top Tox Lists

Name	p-value	Overlap
Mitochondrial Dysfunction	9.76E-20	18.1 % 25/138
Decreases Transmembrane Potential of Mitochondria and Mitochondrial Membrane	6.70E-02	4.2 % 4/96

(c) 2000-2015 QIAGEN. All rights reserved.

INGENUITY® SYSTEMS

Negative Acute Phase Response Proteins	7.71E-02	20.0 % 1/5
Oxidative Stress	1.83E-01	4.1 % 2/49
Recovery from Ischemic Acute Renal Failure (Rat)	2.01E-01	7.1 % 1/14

Top My Lists

Name	p-value	Overlap
175 WvC only (mostly neuronal) molecules	4.77E-06	8.0 % 12/150
90 WvE only (mostly neuronal) molecules	1.09E-04	10.1 % 7/69
66 EvC only (mostly neuronal) molecules	4.74E-02	5.9 % 3/51

Top Analysis-Ready Molecules

Exp Fold Change up-regulated

Molecules	Exp. Value	Exp. Chart
**SLC35D3**	↑ 7.436	
**ZDHHC17**	↑ 6.049	
WWP2	↑ 5.844	
**ADD1***	↑ 5.557	
ELFN1	↑ 5.470	
**FGFR3**	↑ 5.341	
**ABCB11**	↑ 4.457	
GDPD5	↑ 3.613	
**LAMP5**	↑ 3.531	
WAPAL	↑ 3.406	

Exp Fold Change down-regulated

Molecules	Exp. Value	Exp. Chart
**CAMK2A***	↓ -73122162827.637	
**PPM1L**	↓ -34788667566.644	

(c) 2000-2015 QIAGEN. All rights reserved.

INGENUITY® SYSTEMS

**AVP**	↓ -526440535.887
H2AFY	↓ -102.595
CENPV	↓ -23.080
CDC5L	↓ -18.225
**MOBP**	↓ -14.701
H3F3A/H3F3B	↓ -11.249
**CLDN11**	↓ -10.707
**CPLX3**	↓ -7.681

(c) 2000-2015 QIAGEN. All rights reserved.

INGENUITY® SYSTEMS

INGENUITY PATHWAY ANALYSIS

Analysis Name: NAc-WvC (NS,NR)
Analysis Creation Date: 2015-11-27
Build version: 355958M
Content version: 24718999 (Release Date: 2015-09-14)

Analysis Settings

Reference set: Ingenuity Knowledge Base (Genes Only)

Relationship to include: Direct and Indirect

Includes Endogenous Chemicals

Optional Analyses: My Pathways My List

Filter Summary:

Consider only molecules and/or relationships where

(confidence = Experimentally Observed) AND

(tissues = Amygdala OR Cells not otherwise specified OR Cerebellum OR Brainstem OR Hippocampus OR Medulla Oblongata OR Granule Cell Layer OR Granule cells OR Pituitary Gland OR Microvascular endothelial cells OR Stromal cells OR Other Neurons OR Gray Matter OR Cortical neurons OR Other Nervous System OR Astrocytes OR Parietal Lobe OR Neurons not otherwise specified OR Olfactory Bulb OR Striatum OR Cerebral Ventricles OR Corpus Callosum OR Substantia Nigra OR Thalamus OR Caudate Nucleus OR Trigeminal Ganglion OR Brain OR Nervous System not otherwise specified OR Cerebral Cortex OR Putamen OR Sciatic Nerve OR Purkinje cells OR Adipocytes OR Pyramidal neurons OR Other Stem cells OR White Matter OR Dorsal Root Ganglion OR Endothelial cells not otherwise specified OR Nucleus Accumbens OR Choroid Plexus OR Microglia OR Subventricular Zone OR Ventricular Zone OR Hypothalamus OR Stem cells not otherwise specified OR Spinal Cord)

(c) 2000-2015 QIAGEN. All rights reserved.

INGENUITY SYSTEMS

Top Canonical Pathways

Name	p-value	Overlap
Oxidative Phosphorylation	2.21E-10	11.9 % 10/84
Mitochondrial Dysfunction	2.94E-08	7.2 % 10/138
PXR/RXR Activation	5.56E-03	5.7 % 3/53
Clathrin-mediated Endocytosis Signaling	2.12E-02	2.6 % 4/155
Dolichyl-diphosphooligosaccharide Biosynthesis	2.69E-02	25.0 % 1/4

Top Upstream Regulators

Upstream Regulator	p-value of overlap	Predicted Activation
ZFP36L2	4.17E-04	
ATN1	1.04E-03	
HTT	1.40E-03	
PPARD	4.20E-03	
MTTP	6.54E-03	

Top Diseases and Bio Functions

Diseases and Disorders

Name	p-value	#Molecules
Neurological Disease	4.66E-02 - 1.36E-04	17
Organismal Injury and Abnormalities	4.66E-02 - 1.36E-04	9
Psychological Disorders	3.01E-02 - 6.33E-03	10
Ophthalmic Disease	4.01E-02 - 6.80E-03	2
Skeletal and Muscular Disorders	1.68E-02 - 6.90E-03	8

Molecular and Cellular Functions

(c) 2000-2015 QIAGEN. All rights reserved.

INGENUITY® SYSTEMS

Name	p-value	#Molecules
Cell Morphology	4.66E-02 - 3.00E-06	22
Cellular Assembly and Organization	4.66E-02 - 3.00E-06	19
Cellular Development	4.66E-02 - 3.00E-06	19
Cellular Function and Maintenance	4.66E-02 - 3.00E-06	19
Cell-To-Cell Signaling and Interaction	4.66E-02 - 3.12E-05	18

Physiological System Development and Function

Name	p-value	#Molecules
Tissue Development	4.66E-02 - 2.26E-06	19
Embryonic Development	4.66E-02 - 3.00E-06	10
Nervous System Development and Function	4.66E-02 - 3.00E-06	28
Tissue Morphology	4.66E-02 - 3.00E-06	19
Organ Morphology	4.01E-02 - 3.50E-03	7

Top Tox Functions

Assays: Clinical Chemistry and Hematology

Name	p-value	#Molecules
Decreased Levels of Potassium	6.80E-03 - 6.80E-03	1

Top Networks

ID	Associated Network Functions	Score
1	Cell-To-Cell Signaling and Interaction, Nervous System Development and Function, Cellular Development	19
2	Cell Death and Survival, Lipid Metabolism, Molecular Transport	17
3	Cellular Development, Nervous System Development and Function, Tissue Development	17

(c) 2000-2015 QIAGEN. All rights reserved.

INGENUITY® SYSTEMS

4	Hematological System Development and Function, Immune Cell Trafficking, Inflammatory Response	5
5	Digestive System Development and Function, Lipid Metabolism, Molecular Transport	2

Top Tox Lists

Name	p-value	Overlap
Mitochondrial Dysfunction	2.94E-08	7.2 % 10/138
Nongenotoxic Hepatocarcinogenicity Biomarker Panel	4.53E-03	13.3 % 2/15
PXR/RXR Activation	5.56E-03	5.7 % 3/53
Increases Permeability Transition of Mitochondria and Mitochondrial Membrane	5.31E-02	12.5 % 1/8
Glutathione Depletion - CYP Induction and Reactive Metabolites	5.31E-02	12.5 % 1/8

Top My Lists

Name	p-value	Overlap
175 WvC only (mostly neuronal) molecules	6.98E-05	4.7 % 7/150
66 EvC only (mostly neuronal) molecules	2.94E-01	2.0 % 1/51
90 WvE only (mostly neuronal) molecules	3.76E-01	1.4 % 1/69

Top Analysis-Ready Molecules

Exp Fold Change up-regulated

Molecules	Exp. Value	Exp. Chart
MYRIP	↑ 28319514729.674	
CACUL1	↑ 14050171235.129	
**ABCB11**	↑ 4.069	
**MAP2K5**	↑ 2.976	
STARD5	↑ 2.401	
**IFT57**	↑ 2.366	
**HMGCS2**	↑ 2.298	
**ILK**	↑ 2.275	

(c) 2000-2015 QIAGEN. All rights reserved.

INGENUITY® SYSTEMS

**PRKAB1**	↑ 2.202	
**SLC1A3**	↑ 2.180	

Exp Fold Change down-regulated

Molecules	Exp. Value	Exp. Chart
Aldoart1	↓ -154513977101455.470	
CDC5L	↓ -23.384	
H2AFY	↓ -15.348	
IFT122	↓ -10.311	
**MOBP**	↓ -5.781	
**CLDN11**	↓ -4.472	
GFM2	↓ -4.321	
H3F3A/H3F3B	↓ -4.111	
**RASA3**	↓ -3.192	
SMPDL3A	↓ -2.475	

(c) 2000-2015 QIAGEN. All rights reserved.

INGENUITY SYSTEMS
